# SUR-8 interacts with PP1-87B to stabilize PERIOD and regulate circadian rhythms in *Drosophila*

**DOI:** 10.1371/journal.pgen.1008475

**Published:** 2019-11-11

**Authors:** Yongbo Xue, Joanna C. Chiu, Yong Zhang

**Affiliations:** 1 Department of Biology, University of Nevada, Reno, NV, United States of America; 2 Department of Entomology and Nematology, University of California, Davis, CA, United States of America; Fundacion Instituto Leloir, ARGENTINA

## Abstract

Circadian rhythms are generated by endogenous pacemakers that rely on transcriptional-translational feedback mechanisms conserved among species. In *Drosophila*, the stability of a key pacemaker protein PERIOD (PER) is tightly controlled by changes in phosphorylation status. A number of molecular players have been implicated in PER destabilization by promoting PER progressive phosphorylation. On the other hand, there have been few reports describing mechanisms that stabilize PER by delaying PER hyperphosphorylation. Here we report that the protein Suppressor of Ras (SUR-8) regulates circadian locomotor rhythms by stabilizing PER. Depletion of SUR-8 from circadian neurons lengthened the circadian period by about 2 hours and decreased PER abundance, whereas its overexpression led to arrhythmia and an increase in PER. Specifically SUR-8 promotes the stability of PER through phosphorylation regulation. Interestingly, downregulation of the protein phosphatase 1 catalytic subunit PP1-87B recapitulated the phenotypes of SUR-8 depletion. We found that SUR-8 facilitates interactions between PP1-87B and PER. Depletion of SUR-8 decreased the interaction of PER and PP1-87B, which supports the role of SUR-8 as a scaffold protein. Interestingly, the interaction between SUR-8 and PER is temporally regulated: SUR-8 has more binding to PER at night than morning. Thus, our results indicate that SUR-8 interacts with PP1-87B to control PER stability to regulate circadian rhythms.

## Author summary

Circadian clocks govern daily rhythms in physiology and behavior. Conserved molecular machinery drives circadian clocks among animals. PERIOD is a key pacemaker protein in fruit flies that undergoes a series of post-translational modifications. Several kinases have been identified in destabilizing PER. Here we identify the role of SUR-8 in circadian locomotor rhythms. Depletion of SUR-8 in pacemaker neurons slows down circadian rhythms and reduces PER abundance. Indeed, SUR-8 promotes the stability of PER. Finally we characterize SUR-8 as a scaffold protein to bridge PER and a phosphatase (PP1-87B) together to regulate PER phosphorylation and abundance.

## Introduction

Daily rhythms in physiology and behavior are governed by the circadian clock with a period of around 24 hours. The circadian clock synchronizes with environmental cues, such as light and temperature [1–3], and maintain rhythms even under constant conditions. The molecular clocks are evolutionally conserved among most organisms [4–7]. In *Drosophila*, rhythmic transcription of the genes encoding pacemaker proteins PERIOD (PER) and TIMELESS (TIM) is controlled by activators CLOCK (CLK) and CYCLE (CYC) [4,8–11]. In the early-to-mid night, PER and TIM are predominantly in cytoplasm and form a protein complex that undergo a series of post-translational modifications to delay the protein accumulation [12–14]. In the late night, PER/TIM complex then enters the nucleus to inhibit CLK/CYC activity, thereby repressing *per* and *tim* expression [15,16]. In the early morning, light exposure triggers degradation of TIM and then of PER, which releases the repression activity and restarts of feedback loop [17–19]. This negative feedback loop persists with around 24 hour periodicity under constant darkness (DD), i.e., free-running conditions.

In *Drosophila*, the core clock proteins are expressed in a restricted set of about 150 circadian neurons distributed across the brain. These neurons are organized into neuronal network and are further clustered into discrete groups based on anatomical locations: small and large ventral-lateral neurons (s-LNvs, l-LNvs, and the fifth s-LNv), dorsal-lateral neurons (LNds), and three subgroups of dorsal neurons (DNs1, 2 and 3). Among these neurons, small ventral lateral neurons (sLNvs) expressing the pigment-dispersing factor (PDF) neuropeptide are the master pacemaker neurons as they govern circadian rhythms under constant darkness [20–22].

In order to maintain the proper pace of circadian clock, the abundance of PER is critical. *per*^*0*^ null mutant flies are phenotypically arrhythmic with respect to circadian rhythms of locomotor activity and eclosion [23,24]. PER levels oscillate over 24 hours with accumulation from early evening to late night and drops in daytime [25,26]. The abundance and stability of PER is in large part regulated by a series of post-translational events such as phosphorylation, glycosylation and ubiquitination [27–30]. Several kinases have been extensively studied to control PER stability, including casein kinase 1 (CK1, DBT in flies), CK2, NEMO, and cAMP-mediated protein kinase A [30–33]. However, only a few proteins, mainly phosphatases, such as protein phosphatase 2A and protein phosphatase 1 (PP1), have been identified as positive regulators of PER stability or PER/TIM complex [34,35].

In this study, we identified that the scaffold protein, suppressor of Ras (SUR-8) is a critical component for the regulation of PER stability and circadian rhythms. Depletion of SUR-8 lengthened the circadian period by about 2 hours accompanied with decreased PER abundance in circadian neurons as well as delayed TIM nuclear entry. PER induction in clock neurons can rescue the molecular and behavioral defects in *Sur-8* knockdown flies. SUR-8 promotes PER stability by reducing its phosphorylation. Downregulation of PP1-87B, one of the five catalytic subunits of *Drosophila* protein phosphatase 1 (PP1) in clock neurons recapitulated the behavioral and molecular phenotypes of SUR-8 depletion. Furthermore, we found that SUR-8, PER, and PP1-87B are associated in the same protein complex, and knockdown of *Sur-8* impaired the interaction of PER and PP1-87B. Specifically the interaction between SUR-8 and PER is higher at night during the PER accumulation phase as compared to the morning when PER is degrading. These data indicate that SUR-8 serves as a key determinant of circadian rhythms by regulating PER stability through PP1-87B mediated-dephosphorylation.

## Results

### SUR-8 downregulation lengthens circadian locomotor period

**Fig 1:**
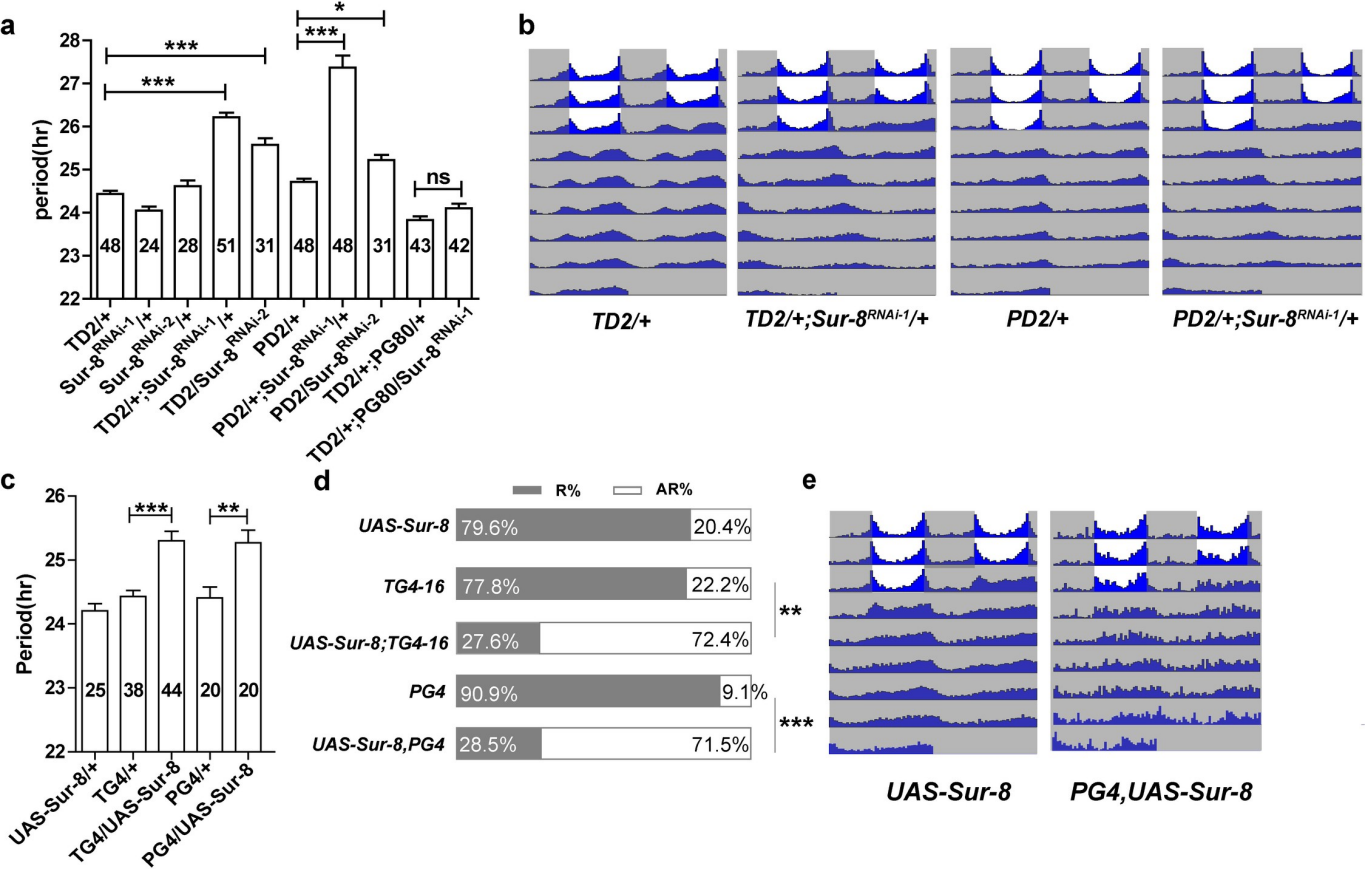
SUR-8 misexpression in circadian neurons leads to disruption of circadian rhythms. **a** The behavioral period of *Sur-8* knockdown in clock neurons.*TD2* stands for *tim-GAL4*, *UAS-dicer2*; *PD2* for *Pdf-GAL4*, *UAS-dicer2*; *PG80* for *Pdf-GAL80*. RNAi-1 indicates BL29557, and RNAi-2 indicates BL60894. The values inside bars represent total flies tested. Error bars indicate SEM. *P < 0.05, ***P < 0.001, and Tukey's Multiple Comparison Test after one-way analysis of variance. **b** Double-plotted actograms showing average activity of *Sur-8* knockdown in all circadian neurons and PDF-positive neurons. White, lights-on; grey, lights-off. **c** Heterozygous SUR-8 overexpression, namely one copy of UAS and GAL4, leads to slightly lengthened behavioral period. *TG4*, *tim-GAL4; PG4*, *Pdf-GAL4*. Error bars indicate SEM. **P < 0.01, ***P < 0.001, Tukey's Multiple Comparison Test after one-way analysis of variance. **d** Homozygous SUR-8 overexpression, namely, two copies of UAS and GAL4, causes severe arrhymicity. *TG4-16*, *tim-GAL4-16*. Error bars indicate SEM. **P < 0.01, ***P < 0.001, Tukey's Multiple Comparison Test after one-way analysis of variance. **e** Double-plotted actograms showing average activity of homozygous SUR-8 overexpression in PDF-positive neurons.

Ras/MAPK has been proposed to affect circadian output in flies [36], we wondered whether Ras signaling affects other function of circadian rhythms. To this end, we harnessed the RNAi pathway to downregulate most Ras signaling related genes in specific clock tissues exploiting the binary GAL4/UAS system. *UAS-Dicer2* was co-expressed with GAL4 drivers to enhance RNAi efficiency [37]. One of the genes that showed the strongest effect is *Sur-8*. Knockdown of *Sur-8* (Sur-8^RNAi-1^) in all clock cells by *tim-GAL4* lengthened circadian locomotor period by 1.8 hours compared to the control. A more dramatic period lengthening, by 2.6 hours, was observed when we restricted SUR-8 depletion in PDF-positive neurons by *Pdf-GAL4* (**Table 1; Fig 1A and 1B**). We are unclear why here *Pdf* driver has stronger effects with Sur-8^RNAi-1^ than *tim* driver since it is a weaker GAL4. However, when we combined Sur-8^RNAi-1^ with *tim-GAL4* or *Pdf-GAL4* to make stable stocks, *tim-GAL4* started to show stronger period lengthening than *Pdf-GAL4* (**Table 1**). Nevertheless, a weaker but significant period lengthening effect (~1.2 hr) was also observed upon downregulation of *Sur-8* using another independent dsRNA line (Sur-8^RNAi-2^) targeting different region of *Sur-8* (**Table 1**), which suggests that effects on circadian period were not due to potential off-target effects. To further confirm this, we performed three experiments. First, we downregulated *Sur-8* by expressing these two dsRNAs simultaneously; we observed an enhanced period lengthening effect (**Table 1**). Second, we inhibited *Sur-8* expression in a genetic deficiency background (Df(3R)ED5780), which lacks of the entire *Sur-8* gene and several neighboring genes. As expected, the circadian period was further lengthened (**Table 1**). Last, we confirmed that transcripts of *Sur-8* were indeed downregulated in the fly heads expressing these two RNAi lines. We did not observe significant knockdown of *Sur-8* in fly heads when we used the *tim*-*GAL4* driver (**S1A Fig**). It is likely that SUR-8 is widely expressed, so specific targeting of *Sur-8* in circadian cells may not be detectable in fly heads by qRT-PCR. To address this possibility, we used *elav-GAL4*, a pan-neuronal driver, for knockdown. Indeed, we found that *Sur-8* mRNA levels were significantly reduced by 75% upon expression of Sur-8^RNAi-1^ (by 30% for Sur-8^RNAi-2^) (**S1B Fig**). In addition, the knockdown efficiency correlated with the severity of the effects on period lengthening.

**Table 1:** Circadian behaviors of *Sur-8* knockdown, overexpression and PER rescue.

Genotype	N	Rhythmic flies (%)	Period (h) ± SEM	Power ± SEM
***Sur-8* down-regulation**				
*tim-GAL4*,*UAS-dicer2 (= TD2)/+*	48	87.5%	24.4 ± 0.08	85.3 ± 2.72
*Sur-8*^*RNAi-1*^*/+ (BL29557)*	24	83.3%	24.0 ± 0.12	59.1 ± 6.45
*Sur-8*^*RNAi-2*^*/+ (BL60894)*	28	92.9%	24.6 ± 0.12	74.7 ± 4.12
*TD2/+; Sur-8*^*RNAi-1*^*/+*	51	74.5%	26.2 ± 0.11	60.0 ± 2.11
*TD2/Sur-8*^*RNAi-2*^	31	96.8%	25.6 ± 0.15	71.9 ± 4.42
*TD2/ Sur-8*^*RNAi-2*^*; Sur-8*^*RNAi-1*^*/+*	55	78.2%	26.8 ± 0.09	70.7 ± 3.49
*TD2/Df*	29	93.1%	25.2 ± 0.09	80.6 ± 4.37
*TD2/+; Sur-8*^*RNAi-1*^*/ Df*	48	69.0%	26.8 ± 0.15	59.4 ± 3.33
*pdf-GAL4*,*UAS-dicer2 (= PD2)/+*	48	85.4%	24.7 ± 0.07	72.9 ± 2.32
*PD2/+; Sur-8*^*RNAi-1*^*/+*	48	72.9%	27.3 ± 0.28	49.9 ± 2.03
*PD2/Sur-8*^*RNAi-2*^	31	87.1%	25.2 ± 0.12	63.8 ± 4.09
*TD2/+; Pdf-GAL80/+*	43	95.3%	23.8 ± 0.08	72.3 ± 4.19
*TD2/+; Pdf-GAL80/Sur-8*^*RNAi-1*^	42	61.9%	24.1 ± 0.11	71.6 ± 4.56
***Stable stock vs fresh cross***				
*TD2/+*	24	100%	24.6 ± 0.14	94.6 ± 5.60
*TD2/+; Sur-8*^*RNAi-1*^*/+ (fresh cross)*	24	87.5%	26.4 ± 0.16	58.3 ± 0.50
*TD2/+; Sur-8*^*RNAi-1*^*/+ (stable stock)*	22	81.8%	26.9 ± 0.16	57.8 ± 5.70
*PD2/+*	32	100%	24.6 ± 0.08	84.1 ± 3.74
*PD2/+; Sur-8*^*RNAi-1*^*/+ (fresh cross)*	30	76.7%	27.5 ± 0.24	48.4 ± 3.99
*PD2/+; Sur-8*^*RNAi-1*^*/+ (stable stock)*	32	62.5%	26.2 ± 0.15	59.0 ± 4.27
***Sur-8* overexpression**				
*UAS-Sur8-Flag*	54	79.6%	24.7 ± 0.17	66.0 ± 4.44
*TG4-16*	18	77.8%	24.1 ± 0.33	52.7 ± 7.96
*UAS-Sur8-Flag;TG4-16*	58	27.6%	26.8 ± 1.23	69.3 ± 3.77
*PG4*, *UAS-Sur8-Flag*	56	28.5%	25.5 ± 0.20	50.6 ± 4.95
*PG4*	55	90.9%	25.0 ± 0.26	68,2 ± 4.16
*UAS-Sur8-Flag/+*	25	84.0%	24.2 ± 0.11	55.9 ± 4.08
*TG4/+*	38	94.7%	24.4 ± 0.10	93.5 ± 4.30
*PG4/+*	20	95.0%	24.4 ± 0.17	57.6 ± 7.75
*TG4/UAS-Sur8-Flag*	44	77.3%	25.3 ± 0.15	43.5 ± 2.70
*PG4/UAS-Sur8-Flag*	20	95.0%	25.3 ± 0.19	69.4 ± 6.79
**Sur-8-EGFP**				
*yw*	63	95.2%	24.3 ± 0.06	94.9 ± 2.96
*Sur-8-EGFP/+*	32	96.9%	24.0 ± 0.05	118.2 ± 3.71
*Sur-8-EGFP*	47	78.7%	24.1 ± 0.09	69.3 ± 3.87
**Behavior at 30°C**				
*TD2/+; tub-gal80ts/+*	88	98.9%	23.5 ± 0.09	79.5 ± 1.96
*TD2/+; tub-gal80ts/Sur-8*^*RNAi-1*^	48	50.0%	25.2 ± 0.15	41.9 ± 3.64
**Behavior at 18°C**				
*TD2/+; tub-gal80ts/+*	32	87.5%	24.2 ± 0.11	71.4 ± 4.63
*TD2/+; tub-gal80ts/Sur-8*^*RNAi-1*^	32	75.0%	24.4 ± 0.24	46.1 ± 3.45
**PER rescue**				
*TD2/+*	39	94.9%	24.1 ± 0.07	86.2 ± 4.63
*TD2/+; Sur-8*^*RNAi-1*^*/+*	48	79.2%	26.0 ± 0.18	50.9 ± 3.48
*TD2/+; UAS-gfp/+*	64	98.4%	24.4 ± 0.06	88.6 ± 3.28
*TD2/+; Sur-8*^*RNAi-1*^*/UAS-gfp*	74	50.0%	26.4 ± 0.22	46.1 ± 3.71
*TD2/+; UAS-per/+*	63	81.0%	24.4 ± 0.11	52.7 ± 3.24
*TD2/+; Sur-8*^*RNAi-1*^*/UAS-per*	65	55.4%	24.1 ± 0.12	45.6 ± 4.57
***per* promoterless**				
*per*^*0*^,*7*.*2-2/; TD2/+*	43	95.3%	27.3 ± 0.08	87.4 ± 4.41
*per*^*0*^,*7*.*2–2; TD2/+; Sur-8*^*RNAi-1*^*/+*	55	52.7%	29.3 ± 0.45	45.7 ± 5.88
*per*^*0*^*; TD2/+; 13*.*2–2*	57	100%	26.6 ± 0.07	97.7 ± 3.19
*per*^*0*^*; TD2/+; Sur-8*^*RNAi-1*^*/13*.*2–2*	55	67.3%	29.7 ± 0.54	36.9 ± 4.26
*TD2/+*	48	95.8%	26.0 ± 0.18	80.0 ± 4.10
*TD2/+; Sur-8*^*RNAi-1*^*/+*	62	50.0%	27.6 ± 0.43	39.7 ± 4.51

To determine whether the circadian defects of *Sur-8* knockdown arise during development or in adulthood, we used the GAL80^ts^ system. GAL80^ts^ is a temperature sensitive repressor element of GAL4 [38]. At high temperature, GAL80^ts^ is inactivated allowing the expression the dsRNA, whereas at low temperature, GAL80^ts^ is active thus blocking GAL4 function. Adulthood-specific downregulation of *Sur-8* lengthened the circadian period by 1.7 hours, whereas restricting SUR-8 depletion to developmental stages gave no phenotype (**Table 1, S1C Fig**). We further tested whether SUR-8 depletion affects the well-characterized projections of ventral lateral neurons [39]. Using an anti-PDF antibody, we did not observe any obvious defects in the dorsal projections, contralateral projections, or optic lobe projections from ventral lateral neurons in *Sur-8* knockdown flies (**S1D Fig**). These data ruled out the possibility that circadian phenotypes of *Sur-8* knockdown result from developmental defects.

Having established that *Sur-8* knockdown affects circadian rhythms we asked whether overexpression of *Sur-8* causes circadian defects by generating and analyzing *UAS-Sur-8-Flag* transgenic lines. While overexpression of *Sur-8* using one copy of *tim-GAL4* or *Pdf-GAL4* generated a slightly lengthened period by 0.9 hours (**Fig 1C, Table 1**), overexpression by two copies of *tim-GAL4* severely impaired circadian rhythmicity (28% rhythmicity vs 80% rhythmicity, **Fig 1D, Table 1**). We observed a stronger period lengthening effect with wide variation in the rhythmic flies with two copies of GAL4 (**Table 1**). In fact, the lengthened period was mainly due to the three outliers that had irregular period between 36.4 and 38 hours (**S2A Fig**). To confirm that the high arrhythmicity in *Sur-8* overexpression was not due to defects in circadian neurons development, we dissected fly brains with *Sur-8* overexpression and stained with PDF and PER antibodies. No obvious defects in major projections of PDF neurons were observed (**S2B Fig**). In addition, all major groups of circadian neurons, such as small and large LNvs, DNs, and LNds were all present (**S2C Fig**). Together, these data suggest that SUR-8 regulates circadian locomotor rhythms in clock neurons.

### SUR-8 is expressed in clock neurons

**Fig 2:**
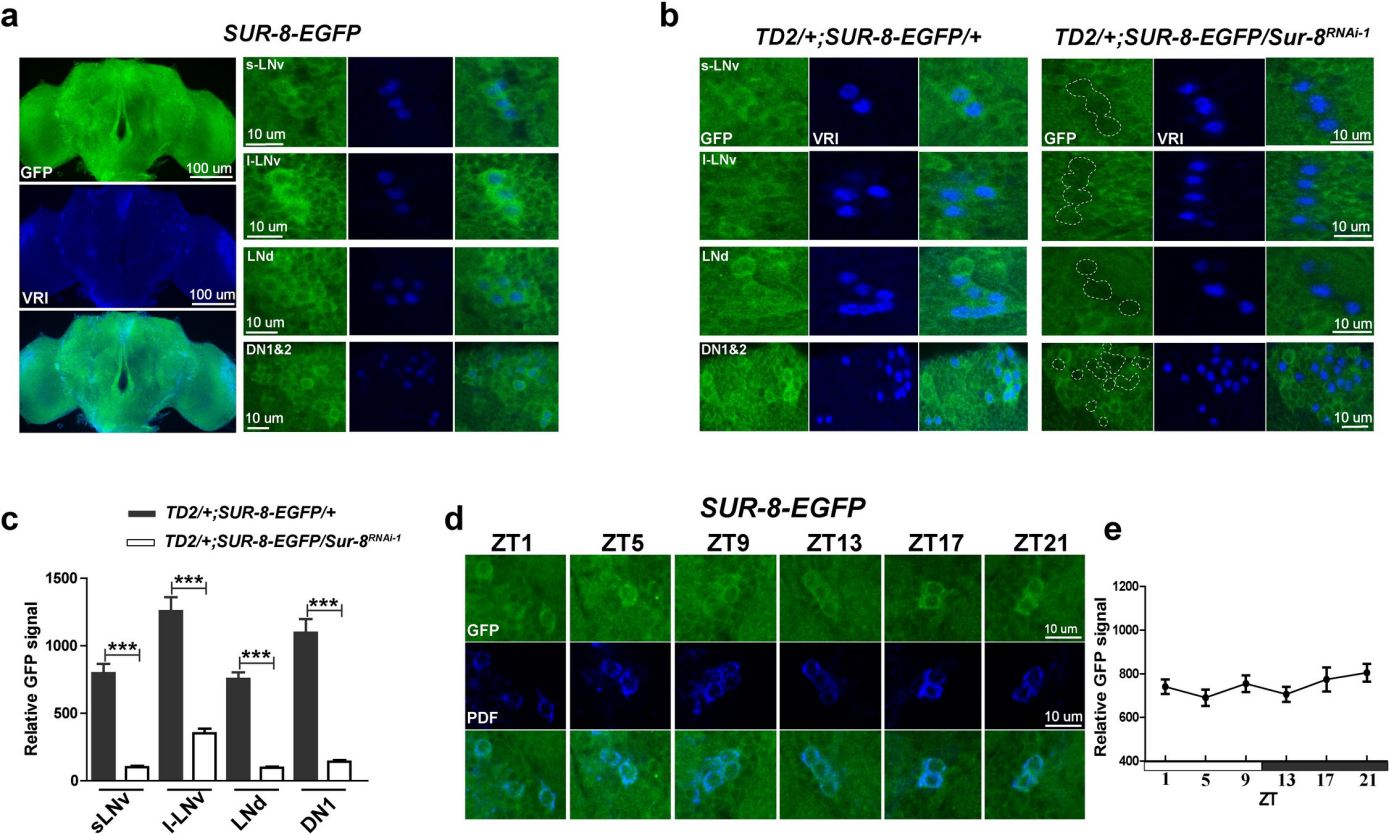
SUR-8 is expressed in clock cells. **a** Representative images of SUR-8-EGFP expression pattern. SUR-8-EGFP flies were entrained for three LD cycles and dissected at ZT15. The spatial expression of SUR-8 was visualized by GFP (green) and clock cells were labeled with anti-VRI (blue) antibodies. **b** Representative images showing *Sur-8* knockdown in SUR-8-EGFP flies. Adult brains were dissected at ZT15 and immunostained with anti-GFP (green) and anti-VRI (blue) antibodies. **c** Quantification of GFP levels in circadian neurons for **b**. Error bars indicate SEM. ***P < 0.001, unpaired t-test. **d** SUR-8 is expressed exclusively in cytoplasm with constant levels in sLNvs during a day. SUR-8-EGFP flies were dissected at indicated timepoints and immunostained with anti-GFP (green) and anti-PDF (blue) antibodies. **e** Quantification of GFP levels in sLNvs for **d**.

We next examined whether SUR-8 is expressed in clock cells. To do so, we used CRISPR/Cas9-mediated homologous recombination to generate an EGFP reporter knock-in into the *Sur-8* locus. The EGFP-coding sequence was inserted immediately upstream of the *Sur-8* stop codon (TGA). SUR-8-EGFP flies were validated by PCR and sequencing (see Material and Methods). We first tested the behavior of homo- and heterozygous Sur-8-EGFP flies and did not observe any period changes (**Table 1**), which suggest that GFP insertion does not affect SUR-8 function. Rhythmicity of homozygous Sur-8-EGFP flies was slightly reduced (78.7% vs 95.2%), but not in heterozygous flies (**Table 1**). We then entrained the SUR-8-EGFP flies for three light/dark (LD) cycles and dissected brains at zeitgeber time 15 (ZT15, 3 hours after lights-off). Samples were immunostained with anti-GFP and anti-VRI antibodies. VRI was used as a marker for circadian neurons. Confocal imaging revealed that SUR-8-EGFP is widely expressed in the fly brain including in clock cells as shown by co-localization of GFP signals with VRI (**Fig 2A**). To confirm the specificity of these GFP signals, we also stained the SUR-8-EGFP in *Sur-8* knockdown with *tim-GAL4*. As expected, GFP signals were severely reduced in most circadian neurons (**Fig 2B and 2C**), which further validates the SUR-8 downregulation by RNAi. We noticed that the knockdown of SUR-8-EGFP in two to three DN1s was not obvious (**Fig 2B**), which might be due to weaker GAL4 expression in these neurons.

To understand whether SUR-8 expression is under circadian control, we determined SUR-8 abundance by monitoring the SUR-8-EGFP levels in sLNvs at different times during a day. SUR-8-EGFP flies were collected at 4-hour intervals and sLNvs were co-labeled with PDF staining. PDF has no oscillation during the day [40]. In the sLNvs, GFP signal remained constant in LD cycles, which indicated that SUR-8 is not under clock regulation. However, we cannot exclude the possibility that GFP might stabilize SUR-8 and abolish potential oscillation. We also observed that SUR-8-EGFP was exclusively localized to the cytoplasm by showing overlap with cytoplasmic PDF signals (**Fig 2D and 2E**).

### SUR-8 impacts the molecular clock in circadian pacemaker neurons

We next examined the effects of *Sur-8* downregulation and overexpression on core clock genes expression. We examined the oscillations of *per*, *tim*, and *Clk* mRNA levels under LD cycles in head extracts. While knocking down *Sur-8* had no effect on the phase of oscillation, it reduced *per*, *tim* transcripts significantly at the time of peak expression (ZT17), and slightly decreased *Clk* mRNA level at ZT21 (**S3 Fig**). When *Sur-8* was overexpressed in circadian tissues, *per*, *tim* transcripts were significantly increased at ZT13, and *Clk* mRNA level was increased at ZT5 (**S3 Fig**).

**Fig 3:**
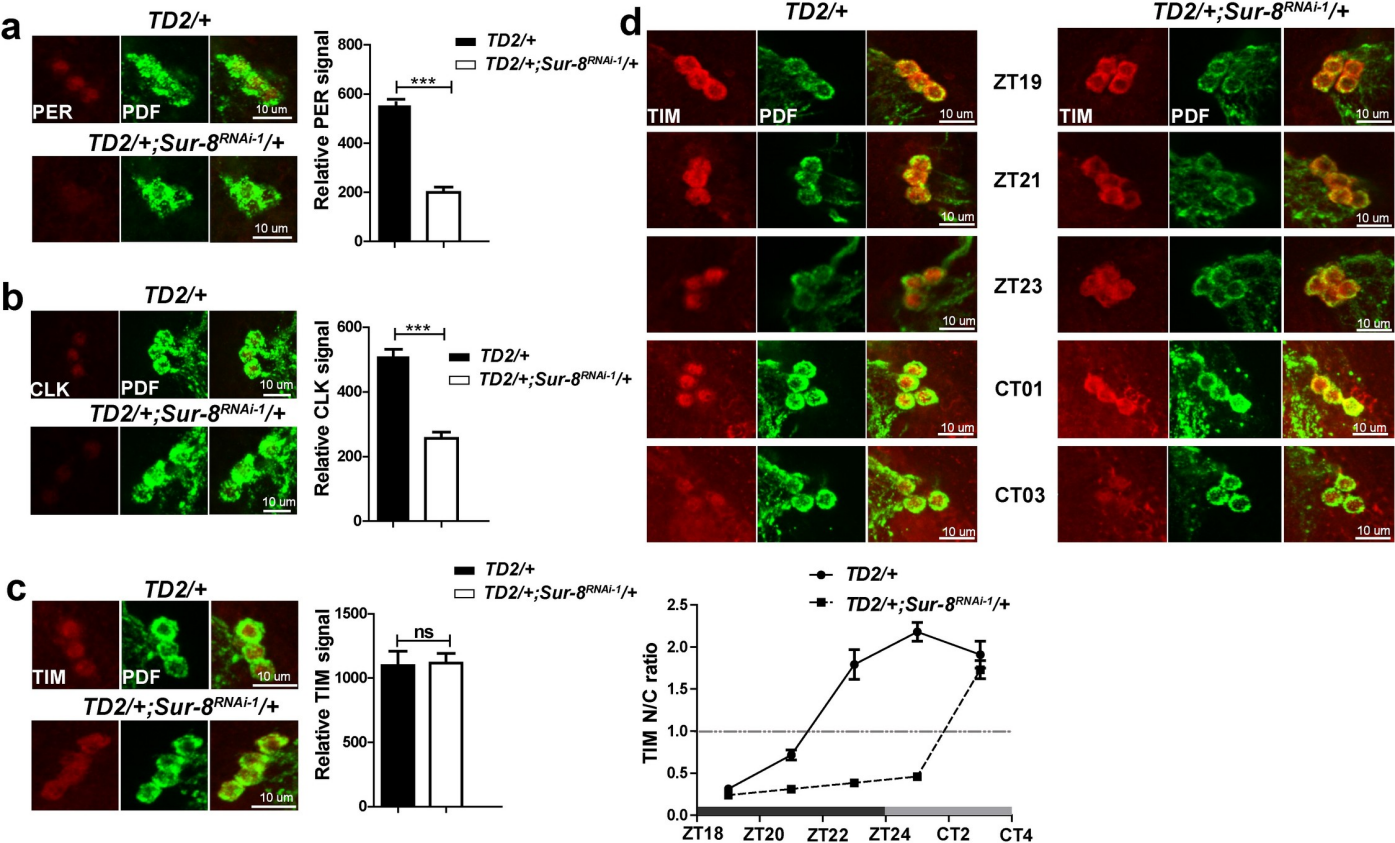
*Sur-8* knockdown disrupts the molecular clock. Brains dissected at the indicated time on day 4 under LD were stained with PDF and one of the clock protein antibodies. Brains were scanned under confocal microscopy and at least 9 brains from each group were quantified using imageJ. **a-c** Representative images of sLNvs immunostained for PDF and PER **a**; PDF and CLK **b**; PDF and TIM **c**. PDF staining is used to label sLNvs. Fly brains were dissected at ZT0 for PER, ZT4 for CLK, ZT0 for TIM, respectively. Quantifications of intensities are shown on the right of corresponding staining. Error bars indicate SEM. ***P < 0.001, ns = non-significant, unpaired t-test. **d** Representative images of sLNvs for TIM (red) and PDF (green) double-staining from late night to early morning to monitor TIM nuclear entry. PDF staining was used as cytoplasm marker of sLNvs. N/C ratio of the y axis of the graph represents TIM levels in nuclear versus cytoplasm. Dotted line in the graph indicates equal TIM amounts in nucleus and cytoplasm.

Inspired by the mRNA changes, we next analyzed core clock proteins levels by immunostaining in sLNvs. We analyzed PER, TIM, CLK, VRI, and PDP1 at their peak levels under LD in *Sur-8* downregulation. Among these core clock proteins, PER and CLK levels were significantly decreased upon *Sur-8* knockdown (**Fig 3A and 3B**), but levels of TIM, VRI, and PDP1 were comparable in SUR-8 depletion and controls (**Fig 3C, S4A and S4B Fig**). We did observe that TIM nuclear entry was delayed in neurons lacking SUR-8 (**Fig 3C**).

We monitored TIM cytoplasm and nuclear accumulation at high resolution at 2-hour intervals from late night (ZT19) to early subjective morning (CT1 and CT3). We used PDF signal as a cytoplasm marker and as a marker for sLNvs location. In the control flies, TIM was observed in the cytoplasm of sLNvs at ZT19, was partially translocated into the nucleus at ZT21, and was observed only in the nucleus at ZT23 (**Fig 3D**). In contrast, TIM appeared to be trapped in the cytoplasm in *Sur-8* knockdown flies from ZT19 to ZT23 (**Fig 3D**). To explore whether TIM was eventually transferred into the nucleus in neurons depleted of SUR-8, we analyzed two additional timepoints at the early subjective morning. We analyzed timepoints in subjective morning because TIM is a light-sensitive protein that starts to get degraded once flies are exposed to light [17,18]. Although TIM was degraded progressively from CT1 to CT3, the majority of TIM protein remained in sLNvs nuclei in control flies at the two subjective morning timepoints. In SUR-8-depleted flies, there was little nuclear accumulation of TIM at CT1, but at CT3 TIM was observed mainly in nuclei (**Fig 3D**). These data demonstrate that *Sur-8* knockdown delays TIM nuclear entry but does not completely block it. Considering that TIM translocates into the nucleus as a complex with PER, we speculate that despite the significant decrease in PER in SUR-8-depleted sLNvs, PER gradually accumulates and eventually reaches a concentration that allows translocation of TIM.

### PER is the primary target of SUR-8

**Fig 4:**
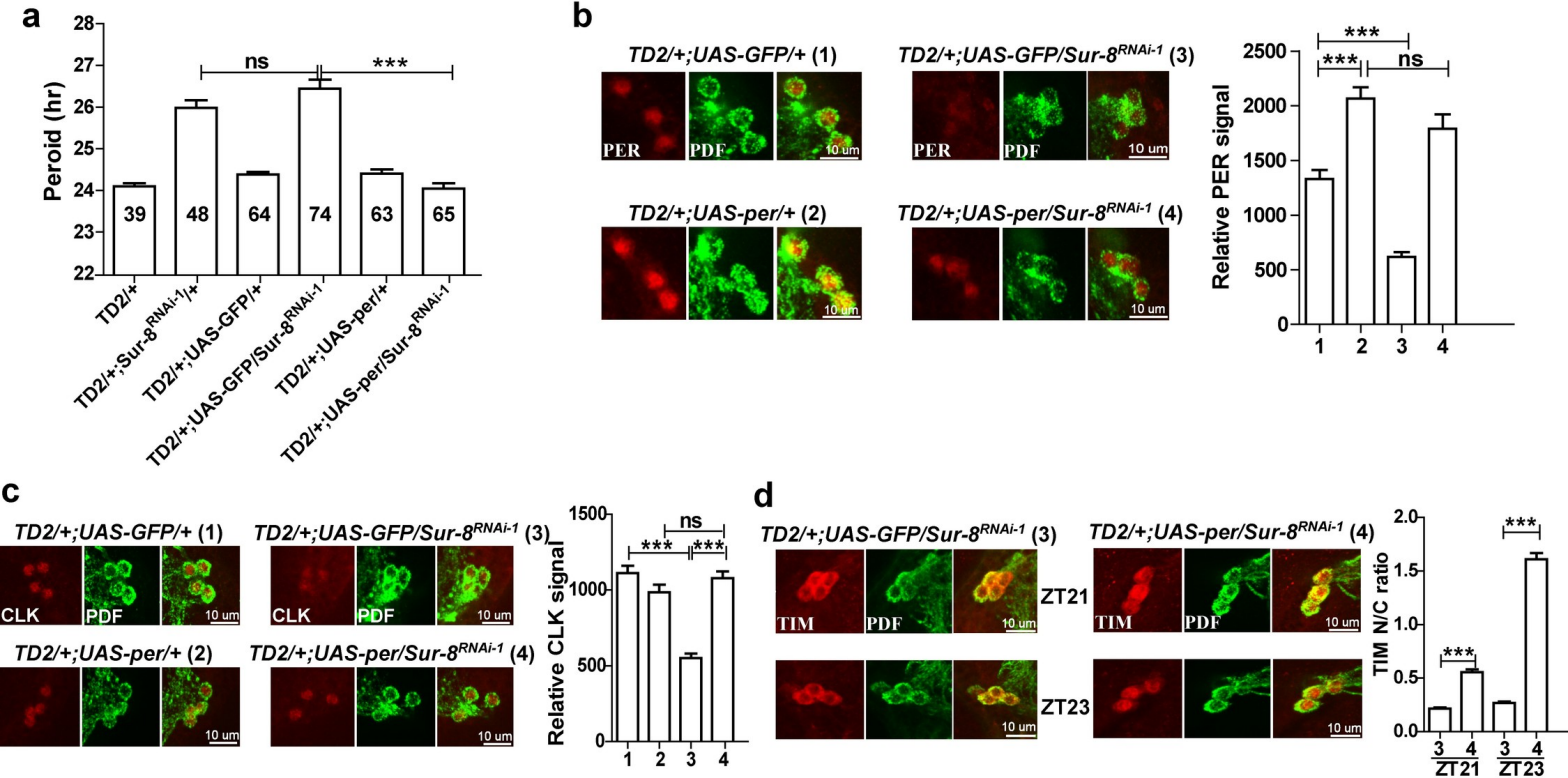
PER is the primary target of SUR-8. **a** PER overexpression rescues the period lengthening in *Sur-8* knockdown. The values inside bars represent total flies tested. Error bars indicate SEM. **P < 0.01, ns = non-significant, unpaired t-test. **b-d** PER restoration in *Sur-8* knockdown flies rescues PER abundance **b**, CLK abundance **c**, and TIM nuclear entry **d**. Immunostaining against PER and CLK was performed at ZT0 and ZT4, respectively. Error bars indicate SEM. ***P < 0.001, ns = non-significant, unpaired t-test.

Although CLK was reduced, TIM, VRI, and PDP1 levels remained unchanged in *Sur-8* knockdown (**Fig 3, S4 Fig**). Since PER exhibited the most dramatic reduction among the pacemaker proteins we detected in *Sur-8* knockdown (**Fig 3**), we hypothesized that PER is the primary target of SUR-8. To address this, we employed the *UAS-per* line, which has been used to rescue *per*^*01*^ [41]. Overexpressing *per* in circadian neurons slightly decreased the rhythmicity (98.4% vs 81% rhythmicity, **Table 1**). Nevertheless, upon overexpression of *per* in *Sur-8* knockdown, we observed a full rescue of circadian period, comparable to the control (**Fig 4A, Table 1**). To exclude the possibility that the rescue was due to competitive binding of UAS, *UAS-GFP* was expressed in *Sur-8* knockdown as a control; in these flies, the circadian period was similar to that of *Sur-8* knockdown flies (**Fig 4A, Table 1**). Encouraged by the behavioral rescue, we next examined whether PER restoration could rescue the abundance of PER and CLK proteins as well as TIM nuclear entry in *Sur-8* knockdown. Our staining and quantification data showed that *per* overexpression restored PER and CLK levels to those observed in control flies (**Fig 4B and 4C**). Likewise, TIM nuclear entry was also rescued when PER was present: TIM was observed only in the nucleus at ZT23 upon overexpression of *per* in the *Sur-8* knockdown background (**Fig 4D**). We therefore conclude that PER is the primary target of SUR-8.

### SUR-8 levels in clock cells affect PER oscillation

**Fig 5:**
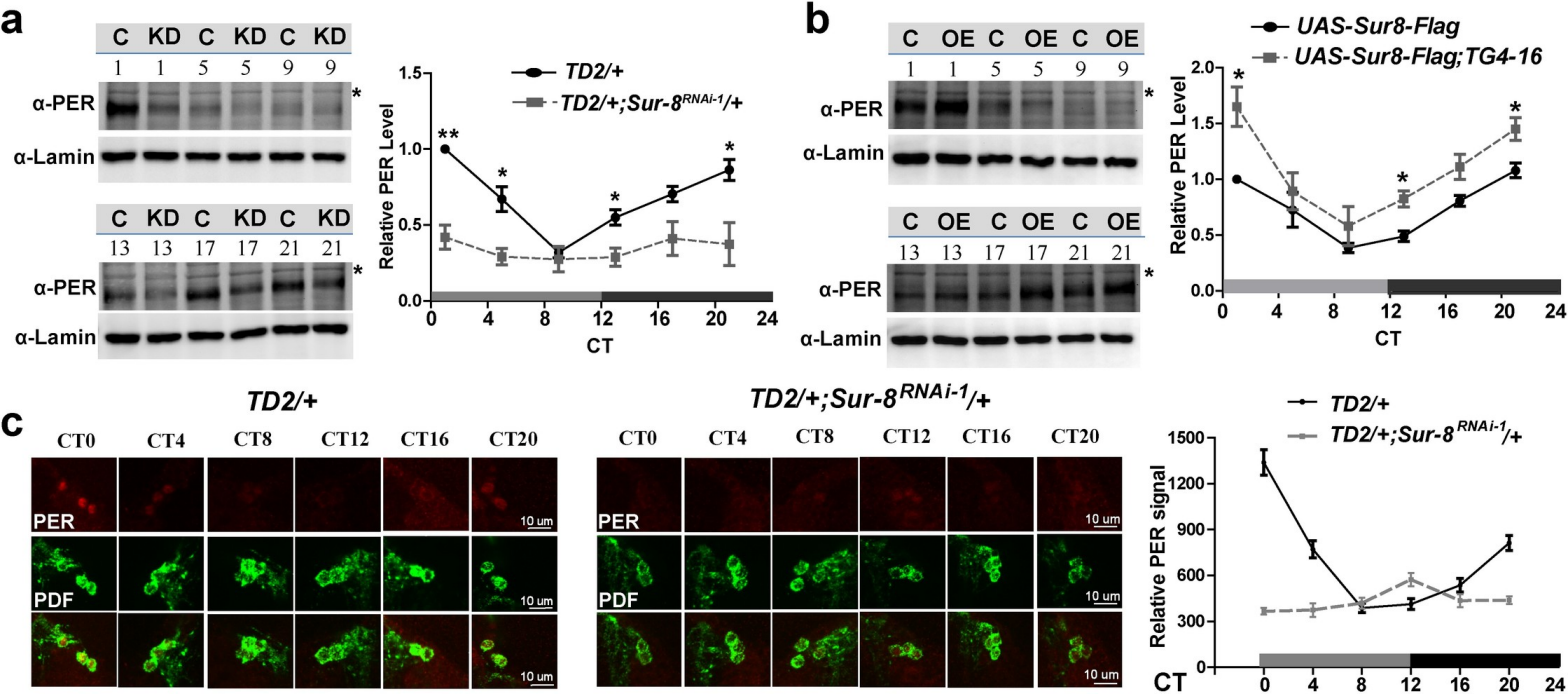
SUR-8 in clock cells regulates PER abundance. **a** Western blots of head extracts from SUR-8 downregulation at indicated time on 1^st^ day of DD. Membranes were probed with anti-PER and anti-Lamin antibodies. Lamin was used as a loading control. * indicates the nonspecific band. C for control group, *TD2/+;* KD for experimental group, *TD2/+;Sur-8*^*RNAi-1*^*/+*. Quantification of PER levels is shown on the right. PER protein levels are normalized to Lamin. PER levels at CT1 in *TD2/+* is set to1. The quantification curve represents three independent repeats. Error bars indicate SEM. *P < 0.05, **P < 0.01, unpaired t-test. **b** Western blots of head extracts from Sur-8 overexpression at indicated time on 1^st^ day of DD. The band labeled with * is a nonspecific band. C for control group, *UAS-Sur8-Flag*; OE for experimental group, *UAS-Sur8-Flag;TG4-16*. Quantification of PER levels is shown on the right. Error bars indicate SEM. *P < 0.05, unpaired t-test. **c** Immunostaining of sLNvs for PER (red) and PDF (green) on the 5^th^ day in DD. Fly brains were fixed, dissected at 4-hour intervals, and imaged under Leica SP8 confocal microscopy. Same parameters were applied to image all fly brains. At each timepoint, at least 15 brains from three independent experiments were quantified. Gray bar, subjective day; dark bar, subjective night.

Having identified PER as the primary target of SUR-8, we decided to characterize PER oscillation over the course of a day in flies depleted of SUR-8. We performed western blots on head extracts on the 1^st^ day of DD. In the wild-type flies, PER proteins undergo daily oscillation in abundance, accumulating at early night, peaking at late night, and declining rapidly at after lights-on (**Fig 5A and 5B**). In *Sur-8* knockdown fly heads, overall PER levels were reduced with dampened oscillation (**Fig 5A**), correlated with the decreased PER in sLNvs, thereby leading to the lengthened circadian period. In contrast, under *Sur-8* overexpression conditions, PER levels were significantly increased relative to levels in wild-type fly heads (**Fig 5B**); however, the oscillation still existed. Additionally, we monitored PER oscillation in the sLNvs by immunostaing on the 5^th^ day of DD. In control flies, PER oscillated with peak expression at late night or early subjective morning, and trough levels at late subjective day or early evening (**Fig 5C**). PER levels in *Sur-8* knockdown flies were significantly lower than controls at most of the time points but, for 4 to 5 days, showed a delayed peak expression at CT8-CT12 due to the lengthened period (**Fig 5C**). Together, the results confirm that SUR-8 affects PER abundance to control circadian rhythms.

### SUR-8 regulates PER at the post-translational level

SUR-8 may affect PER abundance at the transcriptional or post-translational level. The transcript levels of *per* were reduced at ZT13 and ZT17 in *Sur-8* knockdown (**S3A Fig**), suggesting the possibility that SUR-8 regulates *per* transcription. To test this, we performed an *in vitro* assay with a firefly luciferase reporter gene driven by the *per* promoter (*PLO*) [42]. As a control, we co-transfected *PLO* with *pAc-Clk*; this resulted in significant luciferase signal increase indicative of the expected transcriptional activation of the *per* promoter by CLK (**S5A Fig**). By contrast, co-transfection of *PLO* and *pAc-Sur-8-Flag* had no discernable effects on *per* transcriptional activity (**S5A Fig**). Since SUR-8 was unable to activate transcription of the *per* promoter-driven reporter gene, we asked whether the potential transcriptional regulation is CLK-dependent. Co-transfection of *PLO*, *pAc-Clk*, and *pAc-Sur-8-Flag* resulted in luciferase activity comparable to the combination of *pAc-Clk* and *PLO* (**S5A Fig**). Thus, it is unlikely that SUR-8 regulates CLK/CYC mediated *per* transcription directly.

**Fig 6:**
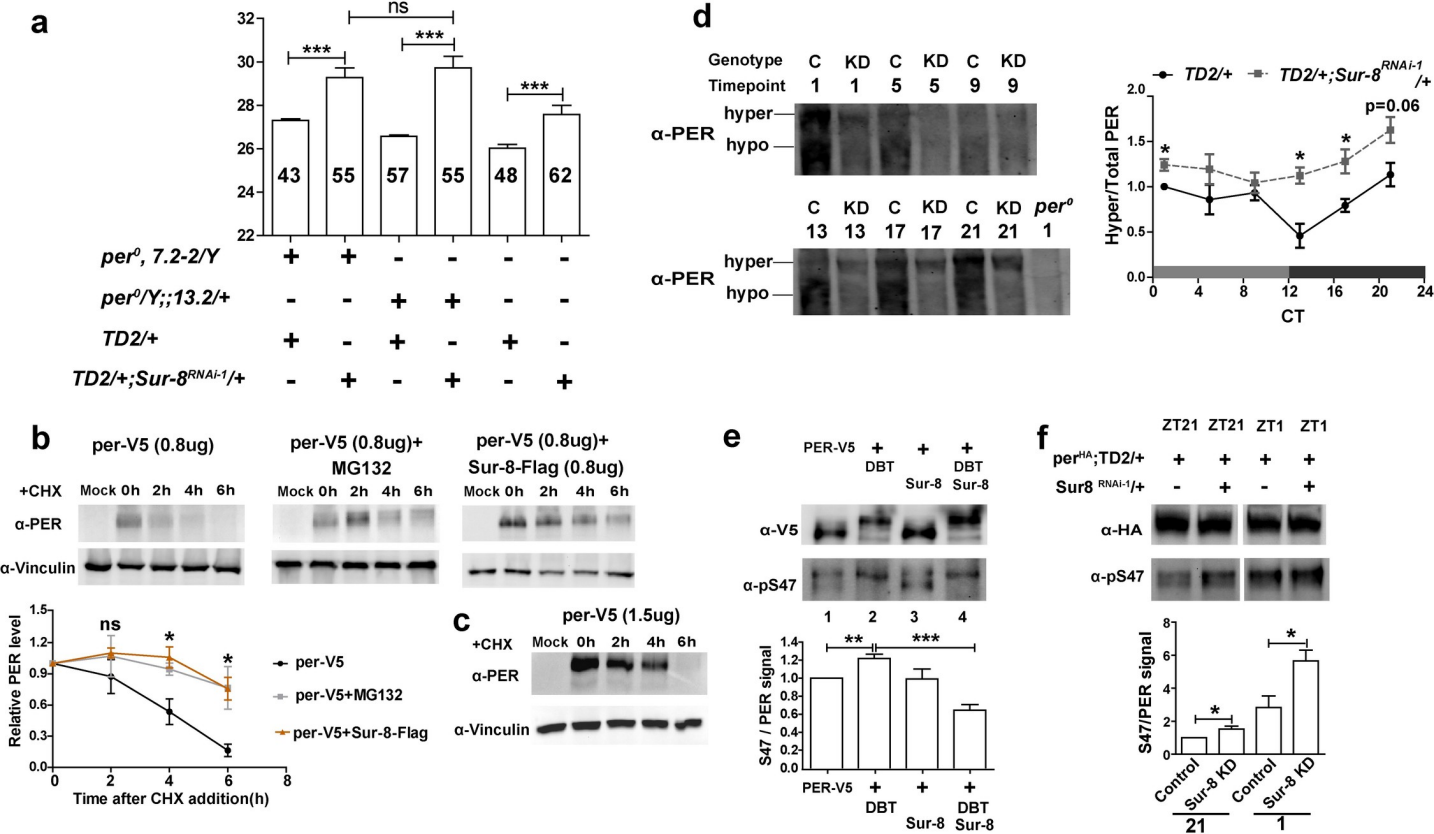
SUR-8 regulates PER stability through phosphorylation. **a** Circadian period of *Sur-8* knockdown in *per* promoter-less line (*per*^*0*^,*7*.*2–2*) or in *per* promoter-containing (*per*^*0*^*;13*.*2*) background. Error bars indicate SEM. ***P < 0.001, unpaired t-test. **b** S2 cells were transiently transfected with *pAc-per-V5* alone (left and middle panels) or along with *pAc-Sur-8-Flag* plasmid (right panel). Cells were treated with CHX after 40 hours transfection to inhibit translation. In the middle panel, S2 cells were also treated with MG132. For imaging of PER bands, short exposure was applied to the right panel (5s) and the other two panels (left and middle) were exposed for 30s. PER protein levels at 0h for each experimental group are set to 1 (n = 4). *P<0.05 as determined by one-way ANOVA with Dunnett’s multiple comparison tests and ns stands for non-significant. **c** S2 cells were transfected with roughly two-fold higher *pAc-per-V5* than **b**. **d** Phos-tag acrylamide gel analysis of PER phosphorylation in *Sur-8* knockdown. C for *TD2/+*; KD for *TD2/+;Sur-8*^*RNAi-1*^*/+*. Fly heads were collected at indicated timepoints on the 1^st^ day of DD. Y axis (right graph) indicates the ratio of hyperphosphorylated PER to total PER. Ratio from control group (*TD2/+)* at CT1 is set to 1. Error bars indicate SEM. *P < 0.05, unpaired t-test. **e** PER S47 phosphorylation analysis in S2 cells with phospho-specific pS47 antibody. Cells were transfected with *pAc-per-V5* together with *pMT-dbt* or *pAc-Sur-8-Flag* or both. DBT expression was induced with CuSO_4_ for 20 hours, and PER proteins were immunoprecipitated with α-V5 antibodies prior to western blots. Loading amounts were adjusted among samples to ensure similar PER levels. The amount of pS47 is presented as a fraction of total PER (n = 5). Error bars indicate SEM. **P < 0.01, unpaired t-test. **f** PER S47 phosphorylation analysis in *Sur-8* knockdown flies with phospho-specific pS47 antibody. Fly heads were collected at ZT21 and ZT1 timepoints. PER-HA proteins were immunoprecipitated with α-HA followed by western blotting analysis. Loading amounts were adjusted among samples to ensure similar PER levels. PER immunoblot at ZT1 was exposed longer than at ZT21, and pS47 immunoblots had same exposure time for both timepoints (n = 3). Error bars indicate SEM. *P < 0.05, unpaired t-test.

Next we examined whether the *per* promoter is required *in vivo* for SUR-8 regulation. We introduced *Sur-8* knockdown into the *per* promoter-less line (*per*^*0*^, *7*.*2*) or the *per* promoter-containing (*per*^*0*^*;;13*.*2*) background [43,44]. In the absence of *per* promotor, *per*^*0*^,*7*.*2* has 0.7 hour longer period than *per*^*0*^*;;13*.*2 flies*. The effects on period lengthening by *Sur-8* knockdown were comparable in the presence (~29.7 hr) or absence of the *per* promoter (~29.3 hr), (**Fig 6A and Table 1**). Taken together, these results suggest that SUR-8-mediated regulation of PER levels is independent of the *per* promoter. Thus the observed changes in *per* mRNA levels upon SUR-8 depletion might result from increased PER-dependent repression of CLK/CYC and/or altered CLK levels.

To investigate whether SUR-8 controls PER abundance through post-translational regulation, we conducted PER half-life analysis in *Drosophila* S2 cells. When *Sur-8* was co-expressed with *per*, we noticed a dosage effect on PER accumulation with increasing levels of the *Sur-8* expression vector (**S5B Fig**). To measure the stability of PER, we transfected S2 cells with respective plasmids at concentrations that resulted in the largest effect on PER accumulation. Cells were harvested at 2-hour intervals after the addition of cycloheximide (CHX) to inhibit translation. S2 cells treated with MG132 were used a positive control [45]. Consistent with prior work [46], we found the half-life of PER proteins was approximately 6 hours (**Fig 6B, left panel**). The half-life of PER was longer in cells treated with MG132 (**Fig 6B, middle panel**), indicating that MG132 inhibited proteasome-dependent PER degradation. Co-expression of SUR-8 with PER had two effects: PER levels were significantly increased and PER proteins were still relative stable 6 hours after the addition of CHX (**Fig 6B, right panel**). The increased PER levels were consistent with *in vivo* results when SUR-8 was over-expressed in clock cells (**Fig 5B**). In the presence of SUR-8, the half-life of PER was similar to the half-life in the presence of MG132, indicating that SUR-8 may stabilize PER by inhibiting its degradation. To rule out the possibility that stabilized PER with SUR-8 co-expression was due to increased baseline levels, we transfected S2 cells with around two-fold higher amounts of *per* expression plasmid; we did not observe a change in PER half-life (**Fig 6C**). We thus concluded that SUR-8 regulates PER at the post-translational level.

### *Sur-8* knockdown enhances phosphorylation of PER

Considering that PER stability is to a large degree regulated by phosphorylation, we monitored PER phosphorylation levels in *Sur-8* knockdown. In order to enhance the separation of PER phosphorylated isoforms, we employed a manganese phos-tag acrylamide gel [47]. Phos-tag western blots of control fly samples had two PER bands: a slower migrating hyperphosphorylated PER band that peaked during late night to early morning and a faster migrating unphosphorylated or hypophosphorylated PER band that peaked at early night (**Fig 6D**). In head extracts of *Sur-8* knockdown flies, the upper band still oscillated but the intensity was weaker compared to the control, and the lower band was present at dramatically lower levels compared to that observed in the control (**Fig 6D**). To accurately represent the changes in PER phosphorylation status, we quantified the band intensities of hyperphosphorylated PER to total PER. We observed that hyperphosphorylated PER increased from early night to early morning in *Sur-8* knockdown flies when compared to control, suggesting that SUR-8 functions to slow down PER hyperphosphorylation.

We also analyzed fly head extracts for PER phosphorylation at serine 47 (pS47) using a phospho-specific antibody [48]. S47 phosphorylation has been reported to be mediated by DOUBLETIME (DBT) and is a crucial step in proteasome-dependent PER degradation [48]. Therefore, we co-transfected *pAc-per-V5* and *pMT-dbt* constructs into S2 cells to recapitulate DBT-dependent PER pS47 phosphorylation [48]. We observed a shift in the PER-V5 band in the presence of DBT expression (**Fig 6E**, α-V5, lanes 2 and 4, and **S7A Fig**). When we normalized pS47 immunoblot signal to total PER-V5, DBT expression significantly increased PER S47 signal (**Fig 6E**, α-pS47, lanes 1 and 2, and **S7A Fig**) as expected. However, co-expression of SUR-8 significantly reduced the relative pS47 intensity even in the presence of DBT (**Fig 6E**, α-pS47, compare lane 4 with lane 2, and **S7A Fig**). Our results therefore indicate that SUR-8 antagonize DBT-dependent phosphorylation of pS47 to slow down proteasomal degradation of PER. To confirm our cellular analysis, we assayed PER pS47 occupancy in *Sur-8* knockdown flies at ZT21 and ZT1. We observed that phosphorylation of S47 was increased compared to flies with wild-type levels of SUR-8 (**Fig 6F,** and **S7B Fig**), which is consistent with the S2 cell results. These data strongly suggest that SUR-8 impact PER stability by regulating phosphorylation of PER, including S47.

### Depletion of *PP1-87b* lengthens circadian period

Since SUR-8 is a scaffold protein that has no known enzymatic activity and since downregulation of SUR-8 increased PER phosphorylation, we reasoned that SUR-8 likely interacts with a phosphatase to regulate PER. One of the potential phosphatases is PP1 [49]. There are five genes that encode catalytic subunits of PP1: *87b*, *96a*, *Y2*, *9c (also known as flapwing*, *(flw))*, and *13c* in flies. Protein phosphatase 19C (Pp4-19C) and D6 (PpD6) are also protein phosphatases that dephosphorylate proteins at serine and threonine residues, but whether they are involved in circadian regulation remains unclear.

**Fig 7:**
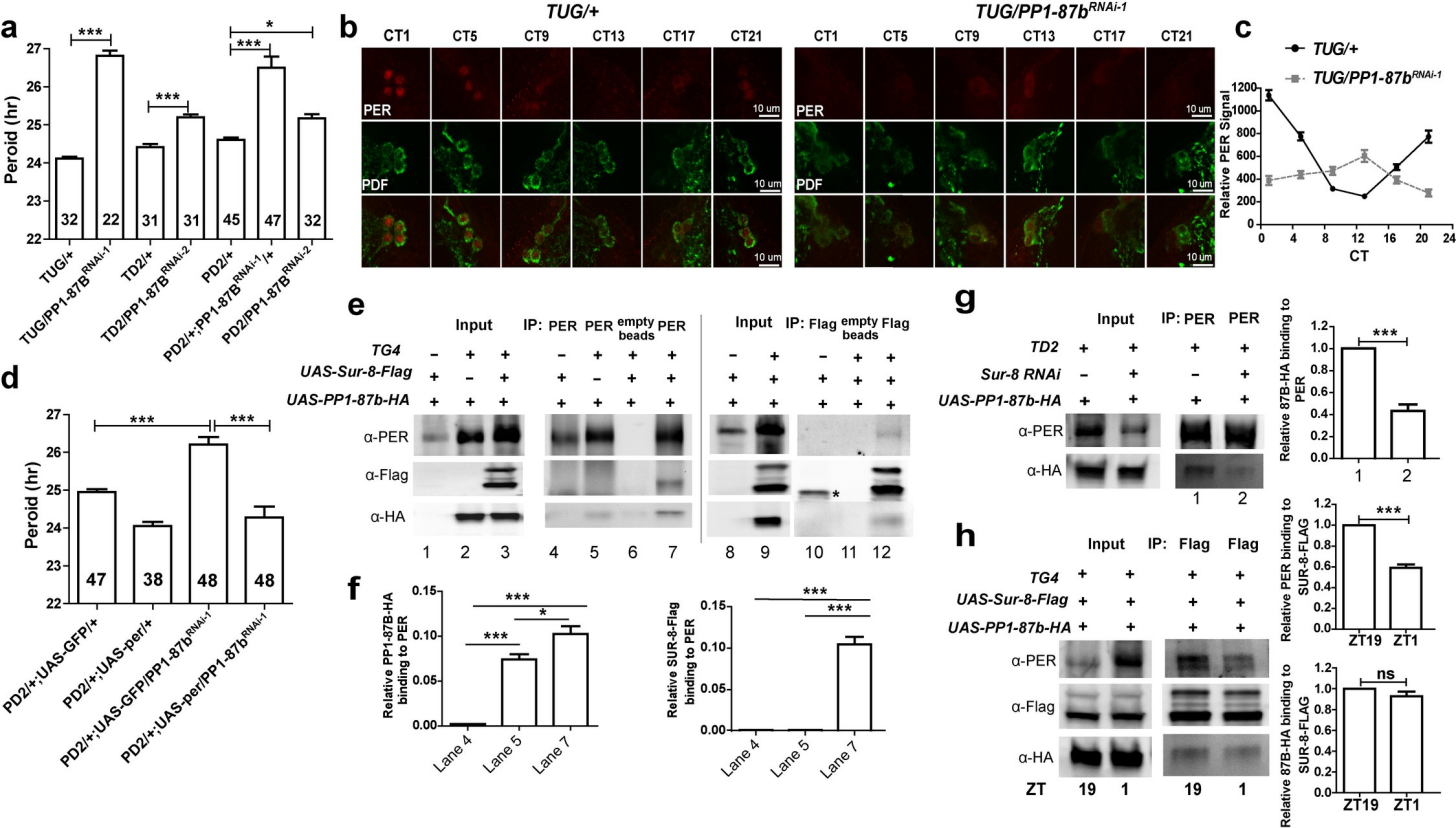
PP1-87B, SUR-8, and PER are in a protein complex. **a** *PP1-87b* knockdown in circadian neurons lengthens circadian period. RNAi-1 (BL32414) expression in *TD2* is lethal, but not in *TUG* (*tim-UAS-GAL4*, a relatively weaker driver). RNAi-2 indicates VDRC35025. Values inside bars indicate tested fly numbers. Error bars indicate SEM. ***P < 0.001, unpaired t-test and Tukey's Multiple Comparison Test. **b** Reduced PER levels and dampened oscillation in *PP1-87b* knockdown. Adult brains were fixed at 4-hour intervals on 5^th^ DD and co-immunostained with anti-PER and anti-PDF antibodies. **c** PER intensities in sLNvs were quantified for **b** from at least eight brains per timepoint. **d** PER overexpression rescues circadian behavior in *PP1-87b* knockdown. Error bars indicate SEM. ***P < 0.001, unpaired t-test. **e** Co-IP analysis of SUR-8, PP1-87B, and PER in fly heads. SUR-8-Flag, PP1-87B-HA, and PER were overexpressed with *tim-GAL4*, and fly heads were collected at ZT19 when PER is mostly cytoplasmic. PER and FLAG antibodies were used to perform Co-IP, respectively. The band at lane 10 against FLAG is a non-specific band, as indicated by *. **f** Quantification of co-immunoprecipitated PP1-87B-HA and SUR-8-Flag in PER IP (n = 3). Error bars indicate SEM. *P < 0.05, ***P < 0.001, unpaired t-test. **g** Co-IP analysis of PER and PP1-87B interaction in the absence of SUR-8 in fly heads at ZT19 (n = 4). Right panel is the corresponding quantification of co-immunoprecipitated PPA-87B-HA against immunoprecipitated PER. Loading amounts were adjusted among samples to ensure similar PER levels. Error bars indicate SEM. ***P < 0.001, unpaired t-test. **h** Co-IP analysis of PER, SUR-8, and PP1-87B interaction at ZT19 and ZT1 (n = 4). Flag antibody was used to pull down protein complex. Right panels are the corresponding quantification of co-immunoprecipitated PER (upper panel) or PPA-87B-HA (lower panel) against immunoprecipitated SUR-8-FLAG. Error bars indicate SEM. ***P < 0.001, ns = non-significant, unpaired t-test.

Therefore, to identify the catalytic subunit of PP1 that interacts with SUR-8 to control PER stability and circadian rhythms, we expressed dsRNAs targeting mRNAs encoding the PP1 catalytic subunits as well as *Pp4-19C* and *PpD6* using *tim-GAL4* and *Pdf-GAL4* (**Table 2**). Overexpression of endogenous PP1 nuclear inhibitor (NIPP1) in circadian neurons lengthened the circadian period (**Table 2**), which is consistent with previous study [35]. Depletion or overexpression of most genes here did not alter the circadian rhythms, even though we could not exclude the possibility of low efficiency in knockdown. Interestingly, depletion of PP1-87B caused a markedly lengthened circadian period (**Fig 7A, Table 2**).

**Table 2:** Circadian behaviors of downregulation of different PP1 isoforms in clock cells.

Genotype	N	Rhythmic flies (%)	Period (h) ± SEM	Power ± SEM
*TD2/+*	31	96.8%	24.4 ± 0.08	74.3 ± 5.26
*TD2/+;PP1-87B*^*RNAi-1*^*/+ (BL32414)*	lethal	-	-	-
*TD2/PP1-87B*^*RNAi-2*^ *(VDRC35025)*	31	90.3%	25.2 ± 0.08	77.4 ± 4.15
*TD2/+;flw RNAi/+ (BL38336)*	15	100%	24.8 ± 0.17	90.7 ± 5.49
*TD2/flw RNAi (BL57022)*	15	100%	24.6 ± 0.17	64.0 ± 5.68
*TD2/PP1α-96A RNAi (BL40906)*	16	100%	25.1 ± 0.11	67.5 ± 4.06
*TD2/PP1α-96A RNAi (BL42641)*	16	56.3%	24.2 ± 0.25	63.4 ± 9.16
*TD2/PP1-Y2 RNAi (BL57236)*	16	100.0%	24.3 ± 0.20	83.6 ± 4.48
*TD2/+;PP1-13C RNAi/+ (BL32465)*	16	100.0%	24.9 ± 0.13	79.2 ± 5.79
*TD2/PP4-19C RNAi (BL57823)*	16	87.5%	24.1 ± 0.19	71.2 ± 6.40
*TD2/+;PP4-19C RNAi/+ (BL38372)*	16	81.3%	24.8 ± 0.21	71.5 ± 6.98
*TD2/+;PP4-19C RNAi/+ (BL27726)*	16	43.8%	24.5 ± 0.17	52.1 ± 7.83
*TD2/PPD6 RNAi (BL62849)*	16	93.8%	24.9 ± 0.12	68.3 ± 6.82
*TD2/+;UAS-NIPP1 (BL23711)*	lethal	-	-	-
*TD2/UAS-NIPP1 (BL23712)*	lethal	-	-	-
*TD2/+;UAS-PP1-13C (BL23701)*	14	71.4%	24.1 ± 0.33	47.3 ± 7.77
*TD2/UAS-I2;UAS-PP1-87B/+ (BL24101)*	15	80.0%	24.7 ± 0.24	59.3 ±10.12
*TD2/+; PP1-87B/+ (BL24098)*	16	93.8%	24.1 ± 0.14	81.9 ± 6.67
*TD2/+; PP1α-96A/+ (BL23700)*	16	68.8%	24.1 ± 0.13	64.7 ± 8.90
*TUG/+*	32	100%	24.1 ± 0.04	101.6 ± 3.95
*TUG/PP1-87B*^*RNAi-1*^*/+ (BL32414)*	22	95.5%	26.8 ± 0.14	58.3 ± 4.79
*TUG/UAS-NIPP1 (BL23711)*	24	58.3%	25.9 ± 0.22	43.7 ± 3.61
*UAS-NIPP1/+;TUG/+ (BL23712)*	10	60.0%	28.4 ± 0.25	66.0 ±12.34
*PD2/+*	45	82.2%	24.6 ± 0.06	67.8 ± 3.54
*PD2/+;PP1-87B*^*RNAi-1*^*/+ (BL32414)*	47	61.7%	26.5 ± 0.29	44.5 ± 3.68
*PD2/PP1-87B*^*RNAi-2*^ *(VDRC35025)*	32	87.5%	25.2 ± 0.11	47.3 ± 3.30
*PD2/+;flw RNAi/+ (BL38336)*	16	75.0%	24.1 ± 0.11	64.1 ± 7.17
*PD2/flw RNAi (BL57022)*	16	93.8%	24.8 ± 0.11	79.7 ± 4.81
*PD2/PP1α-96A RNAi (BL40906)*	16	100%	25.1 ± 0.13	65.5 ± 4.71
*PD2/PP1α-96A RNAi (BL42641)*	15	93.3%	24.2 ± 0.19	70.6 ± 7.30
*PD2/PP1-Y2 RNAi (BL57236)*	16	93.8%	24.5 ± 0.11	81.7 ± 6.45
*PD2/+;PP1-13C RNAi/+ (BL32465)*	16	75.0%	24.5 ± 0.21	61.6 ± 6.32
*PD2/PP4-19C RNAi (BL57823)*	15	93.3%	24.4 ± 0.19	71.9 ± 5.90
*PD2/+;PP4-19C RNAi/+ (BL38372)*	16	93.8%	24.8 ± 0.15	75.6 ± 6.33
*PD2/+;PP4-19C RNAi/+ (BL27726)*	16	81.3%	24.6 ± 0.15	57.9 ± 6.29
*PD2/PPD6 RNAi (BL62849)*	16	93.8%	25.1 ± 0.10	84.9 ± 3.12
*PD2/+;UAS-NIPP1 (BL23711)*	14	85.7%	25.5 ± 0.23	65.2 ± 7.72
*PD2/UAS-NIPP1 (BL23712)*	15	93.3%	26.9 ± 0.21	50.0 ± 5.82
*PD2/+;UAS-PP1-13C (BL23701)*	15	46.7%	23.9 ± 0.16	50.5 ± 11.20
*PD2/UAS-I2;UAS-PP1-87B/+ (BL24101)*	16	87.5%	25.0 ± 0.12	52.1 ± 4.79
*PD2/+; PP1-87B/+ (BL24098)*	18	100%	24.3 ± 0.15	83.2 ± 7.24
*PD2/+; PP1α-96A/+ (BL23700)*	16	56.3%	24.0 ± 0.14	68.1 ± 7.13
**PER rescue**				
*PD2/+; PP1-87b*^*RNAi-1*^*/UAS-per*	48	68.8%	24.3 ± 0.29	50.7 ± 4.50
*PD2/+; PP1-87b*^*RNAi-1*^*/UAS-gfp*	48	64.6%	26.2 ± 0.19	37.7 ± 3.78
*PD2/+; UAS-per/+*	38	73.6%	24.1 ± 0.11	66.7 ± 6.19
*PD2/+; UAS-gfp/+*	47	91.5%	25.0 ± 0.07	75.8 ± 3.70
**Behavior at 30°C**				
*TD2/+; tub-gal80ts/+*	57	100%	23.8 ± 0.05	91.7 ± 2.05
*TD2/+; tub-gal80ts/PP1-87b*^*RNAi-1*^	51	92.2%	25.0 ± 0.07	59.1 ± 2.85

Two independent *PP1-87B* RNAi lines targeting different region of PP1-87b mRNA were employed: RNAi-1 led to a stronger effect and RNAi-2 led to a modest yet significant period lengthening (**Table 2**). We also measured the *PP1-87b* knockdown efficiency by RNAi-1 expressed using *GMR-GAL4*, which drives gene expression in optic lobes [50]. *PP1-87b* mRNA was downregulated by around 50% at ZT1 and ZT13 (**S6A Fig**). In addition, to rule out the possibility of developmental defects in *PP1-87b* knockdown that disrupted circadian rhythms, we performed conditional *PP1-87b* knockdown in adulthood and observed significant period lengthening (**S6B Fig**). These data indicate that PP1-87B is required in adulthood to regulate circadian rhythms.

Next, we assayed changes of core clock proteins upon *PP1-87b* knockdown. Consistent with *Sur-8* knockdown, we observed reduced PER and CLK levels and delayed TIM nuclear entry in *PP1-87b* knockdown flies (**S6C Fig**). Additionally, we tested PER protein cycling under DD upon *PP1-87b* knockdown. PER levels decreased over the course of a day in *PP1-87b* knockdown flies; the small peak in expression at CT13 might be due to the accumulated effects of period lengthening in *PP1-87b* knockdown (**Fig 7B and 7C**). Importantly, PER overexpression rescued the *PP1-87b* knockdown phenotype (**Fig 7D, Table 2**), indicating that PP1-87B regulates circadian rhythms through PER.

### SUR-8 interacts with PP1-87B to regulate PER

As *PP1-87b* knockdown phenocopies *Sur-8* knockdown at both behavioral and molecular levels, we next asked whether the two proteins interact with each other and whether they associate with PER. Since SUR-8 is a cytoplasmic protein, flies were collected at ZT19, a time at which PER is observed in the cytoplasm [34] to interrogate protein interactions. We performed co-immunoprecipitation from head extracts of flies expressing epitope-tagged SUR-8-FLAG and PP1-87B-HA in circadian tissues with anti-FLAG and anti-PER antibodies. Both SUR-8-FLAG and PP1-87B-HA proteins co-immunoprecipitated with PER (**Fig 7E**, lane 7); PP1-87B-HA and PER proteins were also pulled down in reciprocal coimmunoprecipitations using anti-FLAG (**Fig 7E,** lane 12), suggesting that all three proteins interact. To determine whether SUR-8 is necessary for the interaction between PER and PP1-87B, PP1-87B-HA proteins were precipitated in the absence of SUR-8 overexpression. PP1-87B-HA proteins were co-immunoprecipitated with anti-PER antibody without SUR-8 overexpression (**Fig 7E**, lane 5), but this might be because of endogenous SUR-8. Nevertheless, it is noteworthy that we observed relatively more PP1-87B co-immunoprecipitated with PER when SUR-8 was overexpressed (**Fig 7E,** compare lane 5 and lane 7, and **Fig 7F**).

To further test the hypothesis that SUR-8 promotes the interaction between PP1-87B and PER, we performed co-immunoprecipitation with anti-PER in the presence of *Sur-8* knockdown. As expected, depletion of SUR-8 significantly decreased the binding of PER and PP1-87B (**Fig 7G**). Finally, we examined whether the interaction between SUR-8 and PER is temporally regulated. We compared two time points based on the differential subcellular localization of PER: ZT19 (cytoplasm) and ZT1 (nuclear). Using anti-FLAG to pull down SUR-8-FLAG, we detected its interaction with PER or PP1-87B. There was no obvious difference of PP1-87B bound to SUR-8 (**Fig 7H**, lower panel for quantification) between ZT19 and ZT1. However, we observed much stronger PER-SUR-8 interaction at ZT19 when compared to ZT1, even though the later time point had more PER in the input (**Fig 7H,** upper panel). Together, our results indicate that SUR-8 facilitates the interaction between PP1-87B and PER, thus temporally regulates PER stability during its accumulation phase.

## Discussion

In this study, we interrogated the role of SUR-8 in circadian clock regulation. Downregulation of *Sur-8* in clock cells led to a dramatic decrease in PER abundance and a lengthened circadian period in constant darkness. Similar phenotypes were observed upon downregulation of *PP1-87b*, the most abundant catalytic subunit of the phosphatase PP1 [51]. SUR-8 is a scaffold protein that is almost entirely composed of leucine-rich regions [52], which were previously proposed to mediate protein-protein interactions [53]. Our co-immunoprecipitation analysis showed that SUR-8, PP1-87B, and PER form a protein complex. Additionally, SUR-8 acts as a scaffold to stabilize the interaction with PP1-87B with PER, which further stabilizes PER. This explains the reduced PER levels and the increased phosphorylation of PER observed in *Sur-8* knockdown flies.

We proposed that PER is the primary target of SUR-8 based on a few reasons. First, previous studies have shown that in the absence of PER (*per*^*0*^ mutant), the abundance of CLK was reduced both at transcription and translation levels [54,55]. Consistent with these reports, here we observed reduction of *Clk* mRNA and CLK protein in *Sur-8* knockdown. Second, it has been shown that TIM nuclear entry requires the binding of PER protein [13], thus delayed TIM nuclear entry might result from reduced PER levels in SUR-8-depleted neurons. Last, behavioral and molecular restoration of *Sur-8* knockdown upon *per* overexpression further confirmed our hypothesis. We also identified that SUR-8 regulates PER at post-translational level. But why *per* mRNA levels were reduced in *Sur-8* knockdown flies? *per* mRNA levels are controlled by the CLK/CYC-mediated transcriptional activity, which in turn is related to PER phosphorylation levels [56,57]. Here, we found that PER phosphorylation was significantly increased in *Sur-8* knockdown, therefore, CLK/CYC-mediated transcriptional activity might be further repressed and lead to lower expression of *per* mRNA.

Sur-8 is a highly conserved gene among species. Mutation of the mammalian SUR-8 homologue *Shoc2* in mice results in lethality [58], and ubiquitous *Sur-8* knockdown with *actin*-GAL4 driver in flies also led to lethality, indicating the essential role of this conserved factor in regulating development. Nevertheless, conditional knockdown of *Sur-8* in adult flies still gave rise to lengthened period, suggesting the expression of SUR-8 in adulthood is sufficient to maintain the normal circadian behavior. Although SUR-8 is expressed in clock neurons and is required for maintenance of clock function, *Sur-8* transcripts and proteins do not undergo daily oscillation. These results suggest that SUR-8 regulates the circadian clock in a directional manner (i.e., it is not regulated by the circadian clock).

Previous study showed that expression of endogenous PP1 nuclear inhibitor (NIPP1) lengthens circadian period and reduces TIM abundance [35]. We were unable to detect reduced TIM levels in *PP1-87b* knockdown but did demonstrate delayed TIM nuclear entry. That we did not detect a change in TIM levels might be due to the fact that expression of NIPP1 targets all five catalytic isoforms, whereas *PP1-87b*-targeted RNAi reduced levels only of PP1-87B. Our immunostaining data after *PP1-87b* knockdown under both LD and DD cycles suggest that PP1-87B stabilizes PER, consistent with a study in mammalian cells that PP1 dephosphorylates and stabilizes PER2 [59].

Protein phosphatase 2A, was previously identified as positive regulators of PER stability [34]. Although MTS, the catalytic subunit of PP2A dephosphorylates PER in flies, overexpression of MTS shortens the circadian period, which is inconsistent with the highly arrhythmic behavior we observed upon *Sur-8* overexpression. We therefore propose that SUR-8 is unlikely to affect PER stability through PP2A. In our analysis of PP1 catalytic subunits, only *PP1-87b* knockdown resulted in strong circadian defects, consistent with previous reports showing that PP1-87B contributes 80% of the catalytic activity of PP1 [51]. We also noticed *PP1-96a* knockdown showed weak circadian defects: One dsRNA line (BL40906) exhibited slightly lengthened period in *tim-GAL4* and *Pdf-GAL4*; the other non-overlapping line (BL42641) only exhibited reduced rhythmicity in *tim-GAL4*, not in *Pdf-GAL4*. A recent study also found that *PP1-96a* knockdown lengthened circadian period in *tim-GAL4* and *Pdf-GAL4* [60]. Although we cannot exclude SUR-8 might also affect PER through PP1-96A since PP1-87B and PP1-96A are in a complex, PP1-87B is likely the main subunit that interacts with SUR-8.

An intriguing observation in the present study is the interaction between SUR-8 and PER is temporally regulated: a stronger binding at night than early morning. Thus SUR-8/PP1-87B dephosphorylates and stabilizes PER during its accumulation stage. In the early morning (or late night), the binding between SUR-8 and PER decreases, PER is progressively phosphorylated by DBT and CK2 [31,32]. Together, SUR-8 plays a critical role in stabilization of PER through facilitating dephosphorylation by PP1. Considering the structural and functional conservation of SUR-8 and Shoc2, the mammalian homologue, it is possible that Shoc2 regulates circadian rhythms in mammals.

## Materials and methods

### Fly stocks and behavior analysis

Flies were reared on standard cornmeal/agar food at 25°C (except for the GAL80^ts^ experiment) under 12 hour:12 hour LD cycles. The following GAL4 lines were used in this study: *tim-GAL4*, *TD2* (*tim-GAL4*, *UAS-dicer2*), *Pdf-GAL4*, *PD2* (*Pdf-GAL4*, *UAS-dicer2*), *TD2; Pdf-GAL80*, *TD2; tubP-GAL80*^*ts*^, *TG4-16* (3^rd^ chromosome), *ED2* (*elav-GAL4;; UAS-dicer2*), *TUG* (*tim-UAS-GAL4*). All RNAi lines and UAS-overexpression lines were obtained from Vienna *Drosophila* RNAi Center (VDRC) and Bloomington *Drosophila* Center (BL).

Locomotor activity of male flies of 3–4 days old was measured using the Drosophila Activity Monitor (DAM, TriKinetics) with 4 days LD cycles and 7 days DD cycles. Under LD condition, light intensity was controlled at around 500 lux. The data was analyzed using FaasX software, and the average activity actograms were generated using MATLAB with Griffith sleep analysis toolbox.

For adulthood stage *Sur-8* knockdown, *TD2; tubP-GAL80*^*ts*^ flies were crossed with *Sur-8* RNAi flies and reared at 18°C to inhibit GAL4 function. After emergence, the behavior of adult flies was moved to 30°C to induce the expression of *Sur-8* dsRNA. For developmental stage knockdown, flies of the genetic crosses were shifted from 30°C to 18°C.

### Generation of *UAS-Sur-8-Flag* and *Sur-8-EGFP* fly lines

To generate *UAS-Sur-8-Flag* flies, *Sur-8* cDNA was subcloned into pAc-Flag first, and then amplified with attached Flag sequence at C-terminus. Subsequently, the *Sur-8-Flag* fragment was cloned into pUAST vector. The injection of *pUAST-Sur-8-Flag* was completed by Rainbow Transgenic.

The *Sur-8-EGFP* flies were generated by CRISPR/Cas9-induced homology-dependent DNA repair (http://flycrispr.molbio.wisc.edu/). Two sgRNAs were designed to target PAM sequences adjacent to the stop codon:

PAM1: sense: CTTCGGCCGTCCACATCTCACATC
antisense: AAACGATGTGAGATGTGGACGGCC
PAM2: sense: CTTCGTGAGATGTGGACGGCGTT
antisense: AAACAACGCCGTCCACATCTCAC

sgRNAs were cloned into pU6-BbsI-chiRNAs. The donor is composed of homology arm 1 + EGFP + homology arm 2. Homology arm 1 was amplified using the following primers:

F: GTAGCGTGCCGGCCACTTTG
R: CATCTGGCGGTATGGTGAGTGC

EGFP was amplified using the following primers:

F: TCACCATACCGCCAGATGGTGAGCAAGGG-CGAGGAGCT;
R: CCAAACGCCGTCCACATCTTACTTGTACAGCTCGTCCATGC.

Homology arm 2 was amplified using the following primers:

F: GATGTGGACGGCGTTTGGCGC
R: GTAAGCTTGCCTGGAGGAGCC

The donor was further cloned into pCR-Blunt vector (ThermoFisher, k275020). Donor and sgRNAs constructs were co-injected into nos-Cas9 attp2 fly embryos by Rainbow Transgenic.

### Fly brain immunostaining

Flies were fixed in 4% paraformaldehyde in PBST for 1 hour at room temperature before dissection. After dissection 10% normal goat serum in PBST was used to block fly brains at room temperature for 1 hour. Primary antibodies were used as following:

anti-mouse PDF, 1:400 (Developmental Studies Hybridoma Bank), rabbit PER, 1:1500, guinea pig TIM, 1:100, anti-rabbit PDP1, 1:400, guinea pig anti-VRI, 1:10,000, guinea pig anti-CLK, 1:2500. Secondary antibodies were obtained from Jackson ImmunoResearch and were diluted in 1:200 with PBST. Fly brains mounted on slides were imaged on a Leica SP8 confocal microscope and were quantified using NIH ImageJ.

### RNA extraction and qRT-PCR analysis

Total RNA was extracted from fly heads and then subject to reverse transcription using superscript III (Thermo Fisher). Quantitative real-time PCR was carried out using SYBR Master Mix (Thermo Fisher). The following gene specific primers were used:

*per* forward primer (5’-GACTCGGCCTACTCGAACAG-3’)
*per* reverse primer (5’-CGCGACTTATCCTTGTTGCG-3’)
*tim* forward primer (5’-ATGGACTGGTTACTAGCAACTCC-3’)
*tim* reverse primer (5’-GGTCCTCATAGGTGAGCTTGT-3’)
*Clk* forward primer (5’-GCCTCGGAAAGTATTACCTCCC-3’)
*Clk* reverse primer (5’-CCATCTCATAGGCCAGGTCATA-3’)
*rpl32* forward primer (5’-CCGCTTCAAGGGACAGTATC-3’)
*rpl32* reverse primer (5’- ACGTTGTGCACCAGGAACTT-3’)
*Sur-8* forward primer (5’-CCCCAGCACGGTAAAGGAG-3’)
*Sur-8* reverse primer (5’-GGCGGAAGTTGACCGATCTT-3’)
*PP1-87b* forward primer (5’-GATCCGGGGACTTTGCTTGAA-3’)
*PP1-87b* reverse primer (5’-GAGGAACAGGTAATTCGATTCCG-3’)

### Western blots, PER pS47, and co-immunoprecipitation analysis

The fly head and S2 cell extracts were prepared using protein lysis buffer (1X HEPES-Trion lysis buffer from Boston Bioproducts, 10% glycerol, 1 mM DTT, 0.4% NP-40, 1X protease inhibitor and 1X phosSTOP from Sigma Aldrich, and 0.5 mM PMSF). The protein concentration was determined by Bradford assay, and equal amounts of protein (30–50 μg) were loaded into a 6% acrylamide gel. After electrophoresis, proteins were transferred to PVDF membrane in a semi-dry manner, and probed with guinea pig anti-PER antibody or anti-V5-tagged PER (1:4000, Thermo Fisher). For fly head extracts, blots were probed with anti-Lamin as loading control (1:100. Developmental Studies Hybridoma Bank) and anti-vinculin was used as loading control for S2 cell extracts (1:200, Santa Cruz Biotechnology). Horseradish peroxidase-conjugated secondary antibodies (Jackson ImmunoResearch) were diluted 1:5,000. Blots were visualized using Femto ECL (Genesee) reagent under Chemidoc imaging system (Bio-Rad). Protein band intensities were quantified using ImageLab.

Samples were extracted with EDTA-free lysis buffer for phos-tag experiment. The phos-tag 6% acrylamide gels were supplemented with 5 μM phos-tag (Wako, cat# 304–93526) and were run for 1 hour at 60 volts and 120 volts for 3–3.5 hours until 70KD protein ladder reached bottom. After electrophoresis, gels were washed once with 50 ml transferring buffer supplemented with 1 mM EDTA for 15 min, and then washed once using transferring buffer without EDTA for 10 min. The following steps are same as described in regular western blots.

To perform PER pS47 analysis *in vivo*, *per*^*HA*^ flies were crossed with *TD2/Cyo* or *TD2/cyo; Sur-8*^*RNAi-1*^*/TM6B* flies. Flies were collected at ZT21 and ZT1. Head extracts were prepared from around 250 μl fly heads homogenized with lysis buffer, and further lysed at 4°C for 30 min on a rotator. Protein extracts were then incubated with anti-HA antibody overnight at 4°C followed by adding 60 μl Dyna Protein G beads (Invitrogen) for 2 hours. Binding proteins were eluted by 1 X sample buffer boiled for 5 min at 95°C and were then subjected to western blotting analysis using anti-GP PER (5620, 1:1000) and anti-Rb PER S47 (1:1000).

Flies overexpressing PER, SUR-8-Flag, and PP1-87B-HA and control flies (one without driver, one without PP1-87B overexpression) were collected at ZT19 after entrainment for three days. Proteins were then extracted from around 250 μl of fly heads and processed as above.

### Cell culture experiments

S2 cells were cultured in Schneider’s Insect Medium (Sigma) supplemented with 10% FBS and transfected using Cellfectin (Thermo Fisher) reagent following the manufacturer’s protocol. The DNA plasmids used were pAc-empty, pAc-per-V5, pAc-Clk-V5, pAc-Sur-8-Flag, and PLO.

For PER half-life experiments, 0.8 μg pAc-per-V5 and 0.8 μg pAc-Sur-8-Flag were transiently transfected into S2 cells and incubated for 40 hours. Cells were treated with CHX (10 μg/mL, Sigma) or with CHX and MG132 (50 μM, Sigma) and harvested every 2 hours to measure protein stability. Cell extracts were processed as described for western blots.

For the luciferase reporter assay, S2 cells were transiently transfected with 50 ng PLO and pAc-Renilla and various amounts of pAc-Clk-V5 or pAc-Sur-8-Flag or both. After a 24-hour incubation, luciferase levels were measured using Promega Dual-Luciferase Reporter Assay System. Firefly luciferase levels were normalized to *Renilla* luciferase levels as a transfection efficiency control.

For analysis of S47 phosphorylation, S2 cells were transiently transfected with (1) 0.8 μg pAc-per-V5 alone, (2) 0.8 μg pAc-per-V5 and 0.2 μg pMT-dbt, (3) 0.8 μg pAc-per-V5 and 0.8 μg pAc-Sur-8-Flag, (4) 0.8 μg pAc-per-V5, 0.2 μg pMT-dbt, and 0.8 μg pAc-Sur-8-Flag. At 26 hours after transfection, pMT-dbt was induced with 500 uM CuSO_4_, and after for 20 hours cells were harvested, washed with cold PBS, and lysed at 4°C for 30 min. Protein extracts processed as described for *in vivo* pS47 procedures.

## Supporting information

S1 Fig SUR-8 is required in adulthood to regulate circadian locomotor rhythms. **a** qPCR quantification of *Sur-8* mRNA levels from *Sur-8* knockdown using *tim-GAL4*. Total RNA was isolated from fly heads at indicated timepoints. *Sur-8* mRNA levels were normalized to *rpl32*. *Sur-8* mRNA levels from *TD2/+* at ZT1 are set to 1. **b** *Sur8* transcripts are significantly downregulated in pan-neuronal driver, *ED2*. *ED2* represents *elav-GAL4*, *UAS-dicer2*. Error bars indicate SEM. **P < 0.01, ***P < 0.001, unpaired t-test. **c** Restricted *Sur-8* knockdown in adulthood leads to lengthened circadian period, but not in developmental stage. Error bars indicate SEM. ***P < 0.001, ns = non-significant, unpaired t-test. **d** Intact PDF neural network in *Sur-8* knockdown flies. Blunt arrow, dorsal projection; open arrow, contralateral projection; closed arrow, optic lobes.
(TIF)

S2 Fig *Sur-8* overexpression does not affect the development of circadian neurons. **(a)** Circadian period changes in *Sur-8* overexpression flies. Notably, 3 flies (out of 16 rhythmic flies) showed extreme longer period in *Sur-8* overexpression. Each dot or rectangle represents one single fly. UAS-Sur-8, N = 43; UAS-Sur-8;TG4-16, N = 16. **(b-c)** Representative images of brains with half hemisphere and specific clock neurons groups in *UAS-Sur-8* (**b**), and *UAS-Sur-8;TG4-16* (**c**). Brains were dissected at ZT23, and co-immunostained with PDF and PER antibodies. Blunt arrow, dorsal projection; open arrow, contralateral projection; closed arrow, optic lobes.
(TIF)

S3 Fig mRNA changes of core clock genes in *Sur-8* knockdown and overexpression. *per* mRNA (**a-a’)**, *tim* mRNA (**b-b’**), *Clk* mRNA (**c-c’**) levels were measured with qPCR in *Sur-8* knockdown and overexpression flies. Flies were entrained under LD cycles for 4 days, and were collected on day 5 at 4-hour intervals. Total mRNA was isolated from fly heads. Trough mRNA levels are set to 1. White bar, day; dark bar, night. Error bars indicate SEM. ***P < 0.001, **P < 0.01, *P < 0.05, ns = non-significant, unpaired t-test.
(TIF)

S4 Fig *Sur-8* knockdown has no effects on VRI and PDP1 abundance. **a-b** Representative images of sLNvs for PDF and VRI staining in **a**, or PDF and PDP1 in **b**. Quantifications of intensities are shown on the right of corresponding staining. Green, PDF; red, VRI or PDP1. Fly brains were dissected at ZT15 for **a**, ZT18 for **b**. Error bars indicate SEM. ns = non-significant, unpaired t-test.
(TIF)

S5 Fig SUR-8 does not regulate *per* expression at transcriptional level. **a.** Bioluminescence assays of *PLO-luc* (*per* promoter only). S2 cells transfected with *pAc-Clk* or *pAc-Sur-8-Flag*, or in combination. Firefly luciferase is the reporter of *PLO* and firefly luciferase signals were normalized to *Renilla* luciferase activity. Error bar indicates SEM. ns = non-significant, one-way ANOVA with Tukey’s test. **b.** SUR-8 dosage-dependently affects PER protein accumulation. S2 cells were transiently transfected with different amounts *pAc-Sur-8-Flag*, whereas PER expression plasmids (*pAc-per-V5*) were used at constant levels, 0.8ug. Vinculin was used as loading control. PER levels at the start point is set to 1 in the quantification curve. One-way ANOVA with Tukey’s test was performed to determine statistical difference among groups at *P < 0.05, and ns stands for non-significant.
(TIF)

S6 Fig PP1-87B is required for the clock function in flies. **a** *PP1-87b*^*RNAi-1*^ knockdown efficiency in *GMR-GAL4*, which drives expression in optic lobes. Fly heads were collected at ZT1 and ZT13, and total mRNA was isolated. Error bars indicate SEM. **P < 0.01, unpaired t-test. **b** PP1-87B is indispensible for adulthood clock regulation. Flies were raised at 18°C until eclosion, and the behavior of adult flies were then tested at 30°C. Error bar indicates SEM. ***P < 0.001, unpaired t-test. **c** *PP1-87b* knockdown phenocopies *Sur-8* knockdown in PER reduction (left panel), CLK reduction (right panel), and TIM nuclear entry delay (middle panel). Flies were fixed at ZT0 (PER), ZT4 (CLK), and ZT0 (TIM), respectively. Quantification graphs of corresponding protein are shown below the images. Error bars indicate SEM. *P < 0.05, ***P < 0.001, unpaired t-test.
(TIF)

S7 Fig SUR-8 regulates the interaction of PP1-87B and PER. **a** Additional repeat of (**Fig 6E**). **b** Additional repeat of (**Fig 6F**). **c** Additional repeat of (**Fig 7E**). **d** Additional repeat of (**Fig 7G**). **e** Additional repeat of (**Fig 7H**).
(TIF)
