# Supplementary figures and images for: SUR-8 interacts with PP1-87B to stabilize PERIOD and regulate circadian rhythms in Drosophila
Source: PLoS Genet. 2019 Nov 11;15(11):e1008475. doi: 10.1371/journal.pgen.1008475 (PMC6874087; doi:10.1371/journal.pgen.1008475)

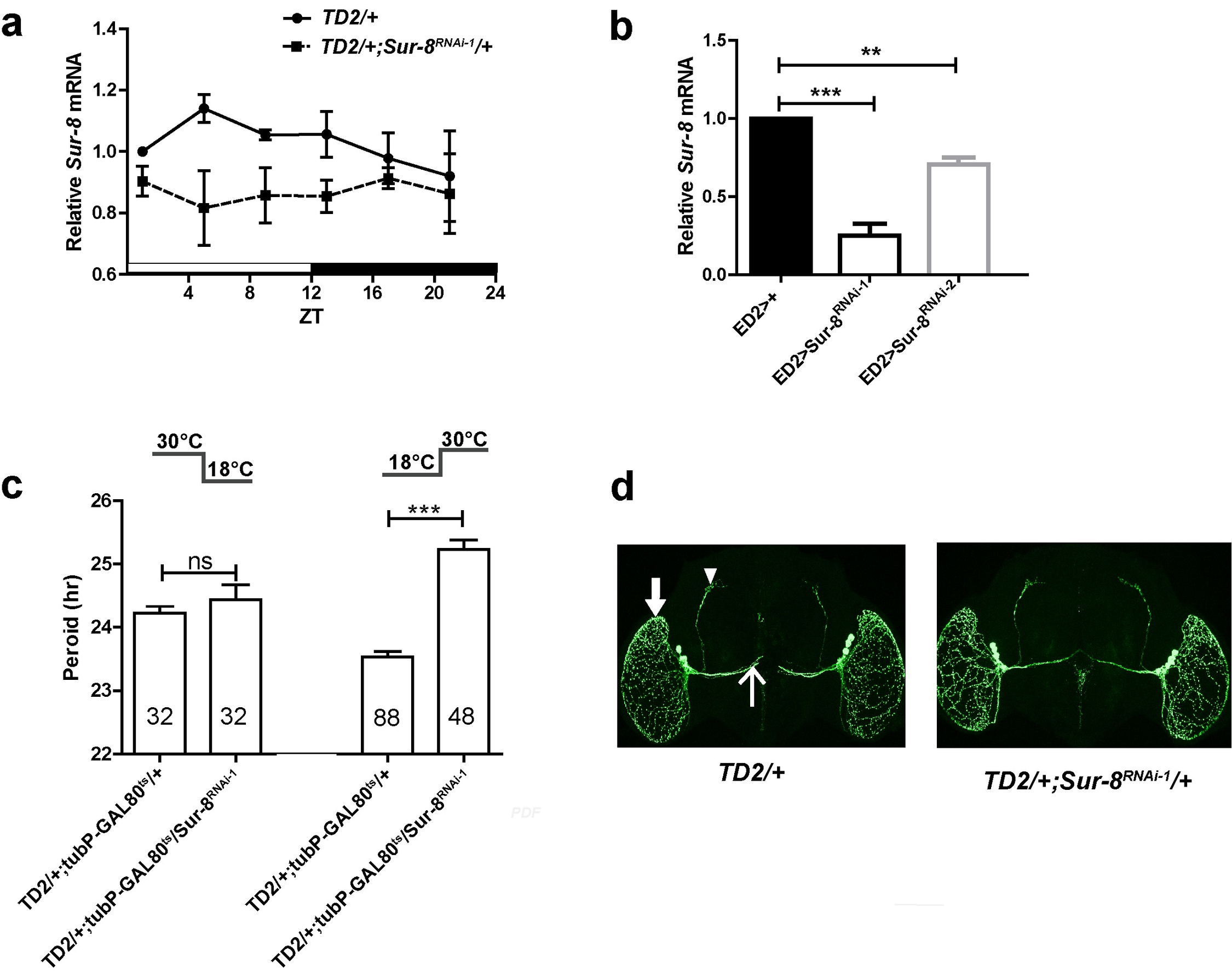

Supplement: S1 Fig — a qPCR quantification of Sur-8 mRNA levels from Sur-8 knockdown using tim-GAL4. Total RNA was isolated from fly heads at indicated timepoints. Sur-8 mRNA levels were normalized to rpl32. Sur-8 mRNA levels from TD2/+ at ZT1 are set to 1. b Sur8 transcripts are significantly downregulated in pan-neuronal driver, ED2. ED2 represents elav-GAL4, UAS-dicer2. Error bars indicate SEM. **P < 0.01, ***P < 0.001, unpaired t-test. c Restricted Sur-8 knockdown in adulthood leads to lengthened circadian period, but not in developmental stage. Error bars indicate SEM. ***P < 0.001, ns = non-significant, unpaired t-test. d Intact PDF neural network in Sur-8 knockdown flies. Blunt arrow, dorsal projection; open arrow, contralateral projection; closed arrow, optic lobes. (TIF) [file pgen.1008475.s001.tif]

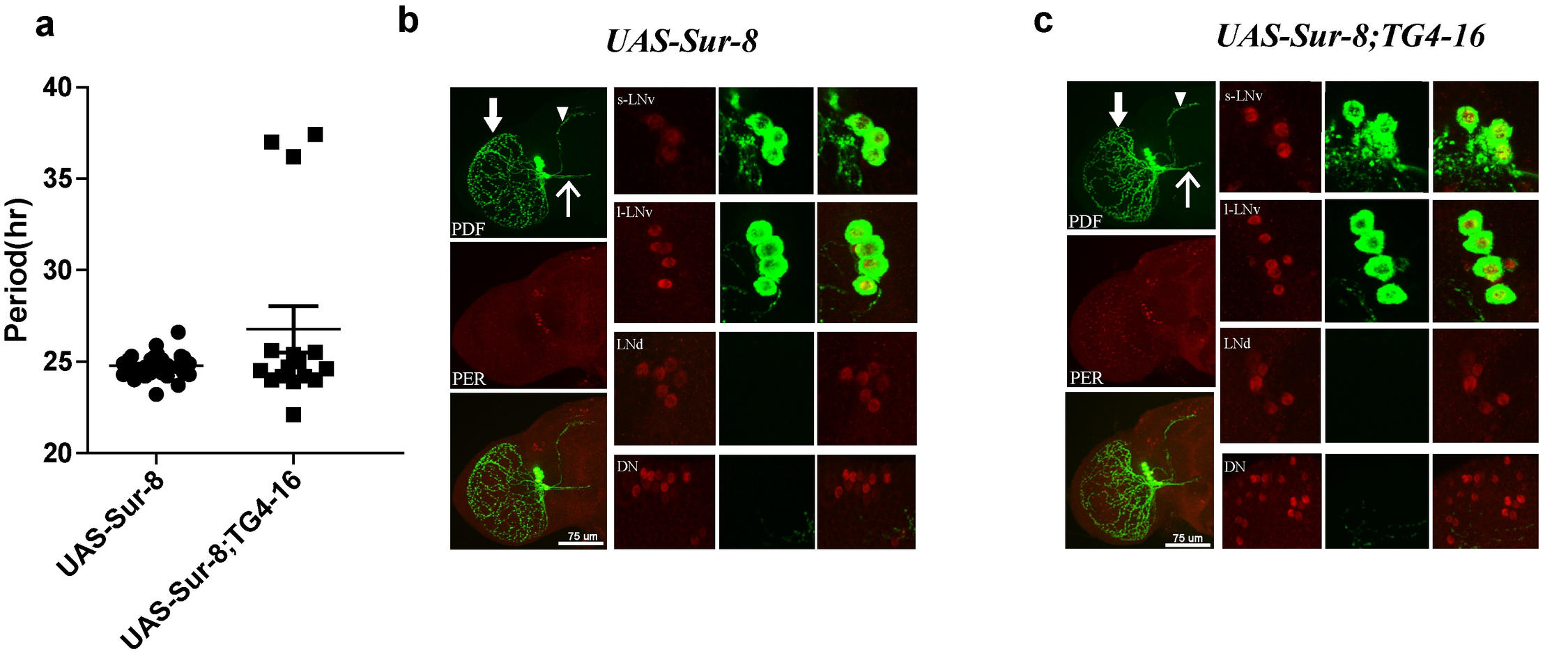

Supplement: S2 Fig — (a) Circadian period changes in Sur-8 overexpression flies. Notably, 3 flies (out of 16 rhythmic flies) showed extreme longer period in Sur-8 overexpression. Each dot or rectangle represents one single fly. UAS-Sur-8, N = 43; UAS-Sur-8;TG4-16, N = 16. (b-c) Representative images of brains with half hemisphere and specific clock neurons groups in UAS-Sur-8 (b), and UAS-Sur-8;TG4-16 (c). Brains were dissected at ZT23, and co-immunostained with PDF and PER antibodies. Blunt arrow, dorsal projection; open arrow, contralateral projection; closed arrow, optic lobes. (TIF) [file pgen.1008475.s002.tif]

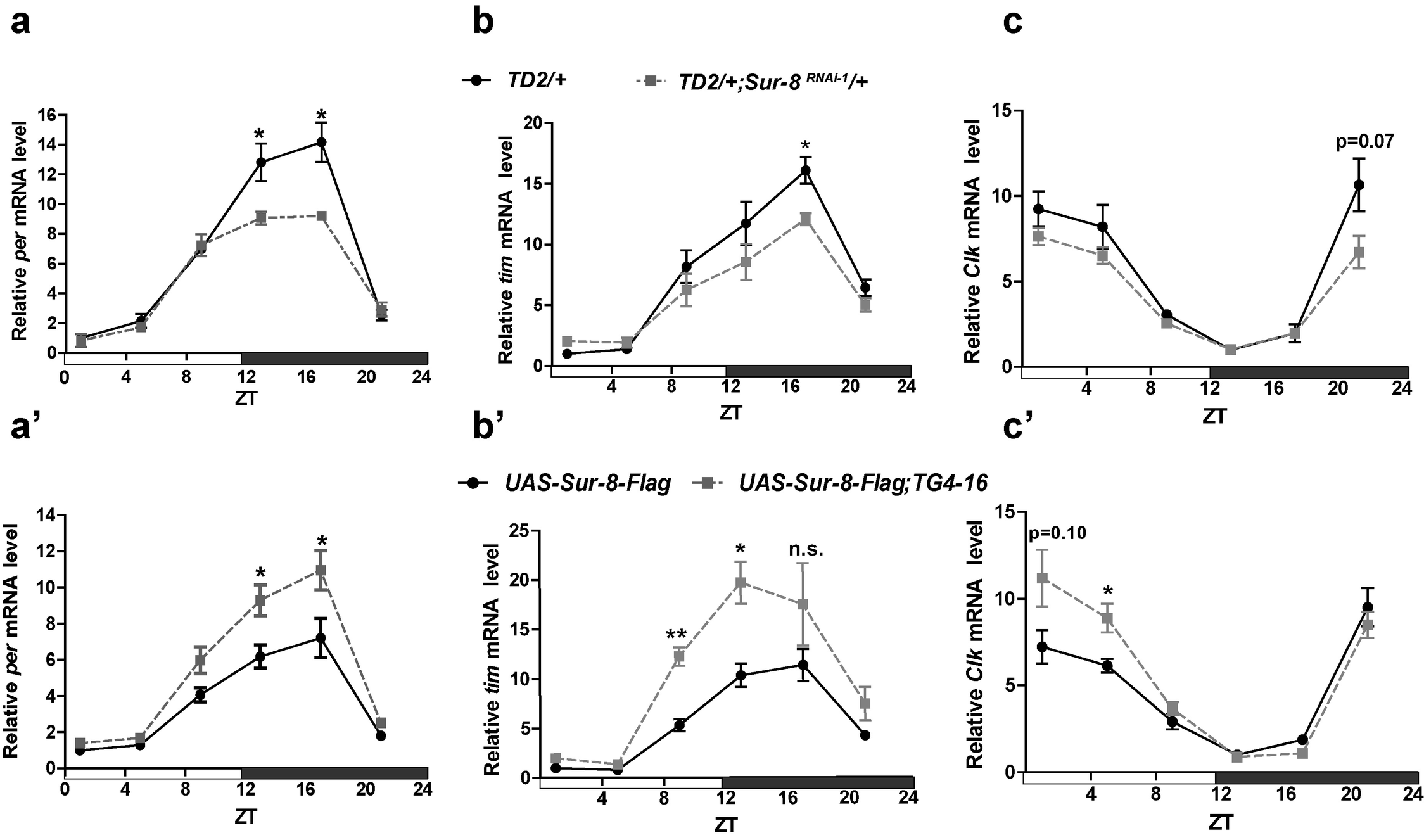

Supplement: S3 Fig — per mRNA (a-a’), tim mRNA (b-b’), Clk mRNA (c-c’) levels were measured with qPCR in Sur-8 knockdown and overexpression flies. Flies were entrained under LD cycles for 4 days, and were collected on day 5 at 4-hour intervals. Total mRNA was isolated from fly heads. Trough mRNA levels are set to 1. White bar, day; dark bar, night. Error bars indicate SEM. ***P < 0.001, **P < 0.01, *P < 0.05, ns = non-significant, unpaired t-test. (TIF) [file pgen.1008475.s003.tif]

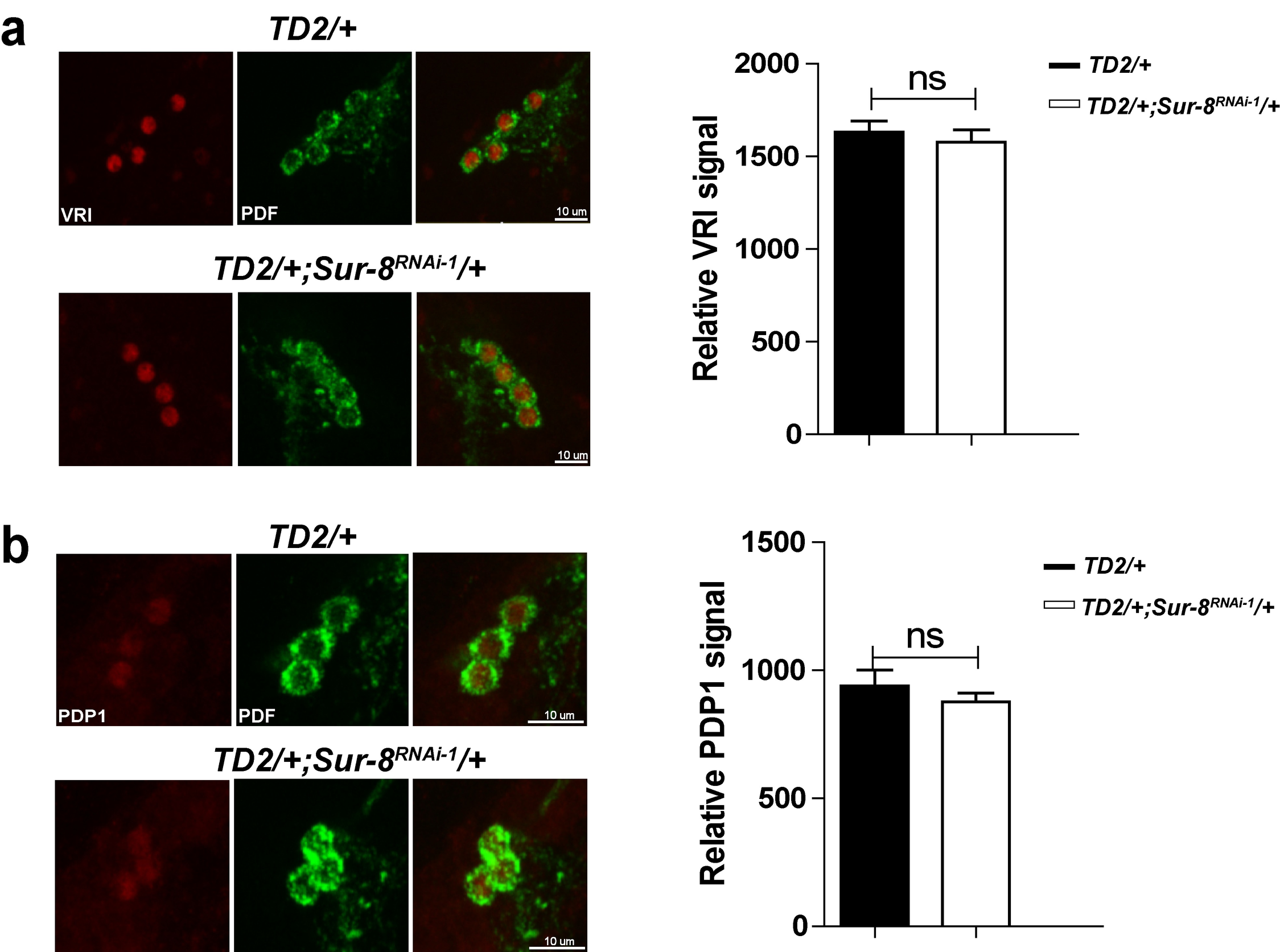

Supplement: S4 Fig — a-b Representative images of sLNvs for PDF and VRI staining in a, or PDF and PDP1 in b. Quantifications of intensities are shown on the right of corresponding staining. Green, PDF; red, VRI or PDP1. Fly brains were dissected at ZT15 for a, ZT18 for b. Error bars indicate SEM. ns = non-significant, unpaired t-test. (TIF) [file pgen.1008475.s004.tif]

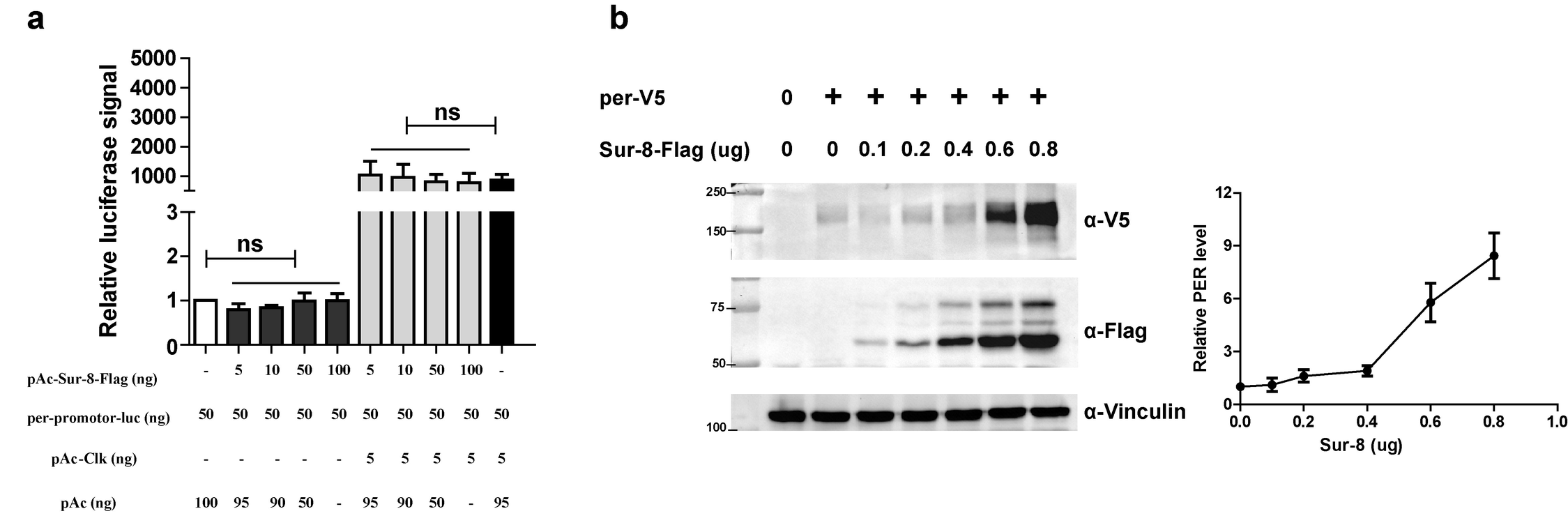

Supplement: S5 Fig — a. Bioluminescence assays of PLO-luc (per promoter only). S2 cells transfected with pAc-Clk or pAc-Sur-8-Flag, or in combination. Firefly luciferase is the reporter of PLO and firefly luciferase signals were normalized to Renilla luciferase activity. Error bar indicates SEM. ns = non-significant, one-way ANOVA with Tukey’s test. b. SUR-8 dosage-dependently affects PER protein accumulation. S2 cells were transiently transfected with different amounts pAc-Sur-8-Flag, whereas PER expression plasmids (pAc-per-V5) were used at constant levels, 0.8ug. Vinculin was used as loading control. PER levels at the start point is set to 1 in the quantification curve. One-way ANOVA with Tukey’s test was performed to determine statistical difference among groups at *P < 0.05, and ns stands for non-significant. (TIF) [file pgen.1008475.s005.tif]

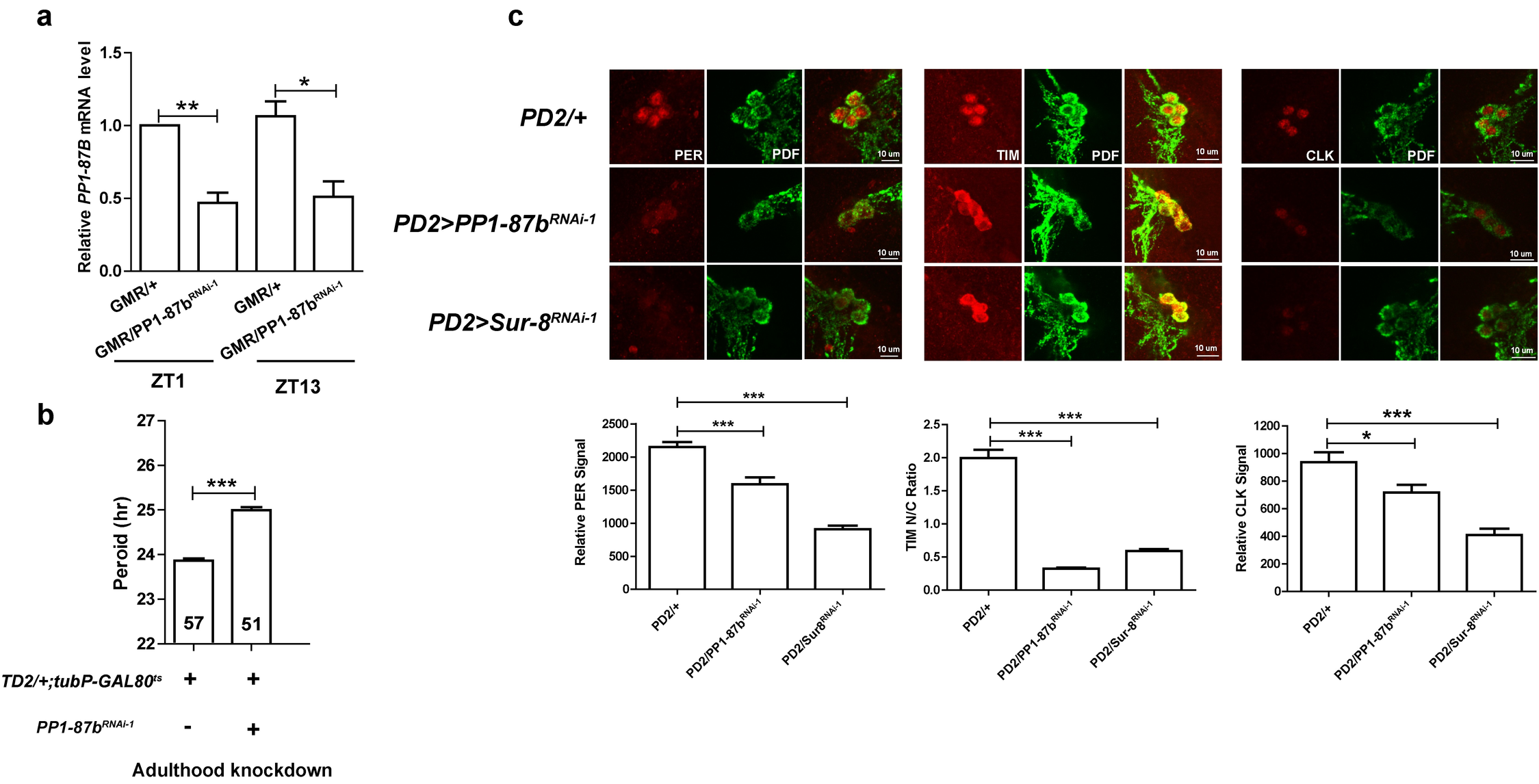

Supplement: S6 Fig — a PP1-87bRNAi-1 knockdown efficiency in GMR-GAL4, which drives expression in optic lobes. Fly heads were collected at ZT1 and ZT13, and total mRNA was isolated. Error bars indicate SEM. **P < 0.01, unpaired t-test. b PP1-87B is indispensible for adulthood clock regulation. Flies were raised at 18°C until eclosion, and the behavior of adult flies were then tested at 30°C. Error bar indicates SEM. ***P < 0.001, unpaired t-test. c PP1-87b knockdown phenocopies Sur-8 knockdown in PER reduction (left panel), CLK reduction (right panel), and TIM nuclear entry delay (middle panel). Flies were fixed at ZT0 (PER), ZT4 (CLK), and ZT0 (TIM), respectively. Quantification graphs of corresponding protein are shown below the images. Error bars indicate SEM. *P < 0.05, ***P < 0.001, unpaired t-test. (TIF) [file pgen.1008475.s006.tif]

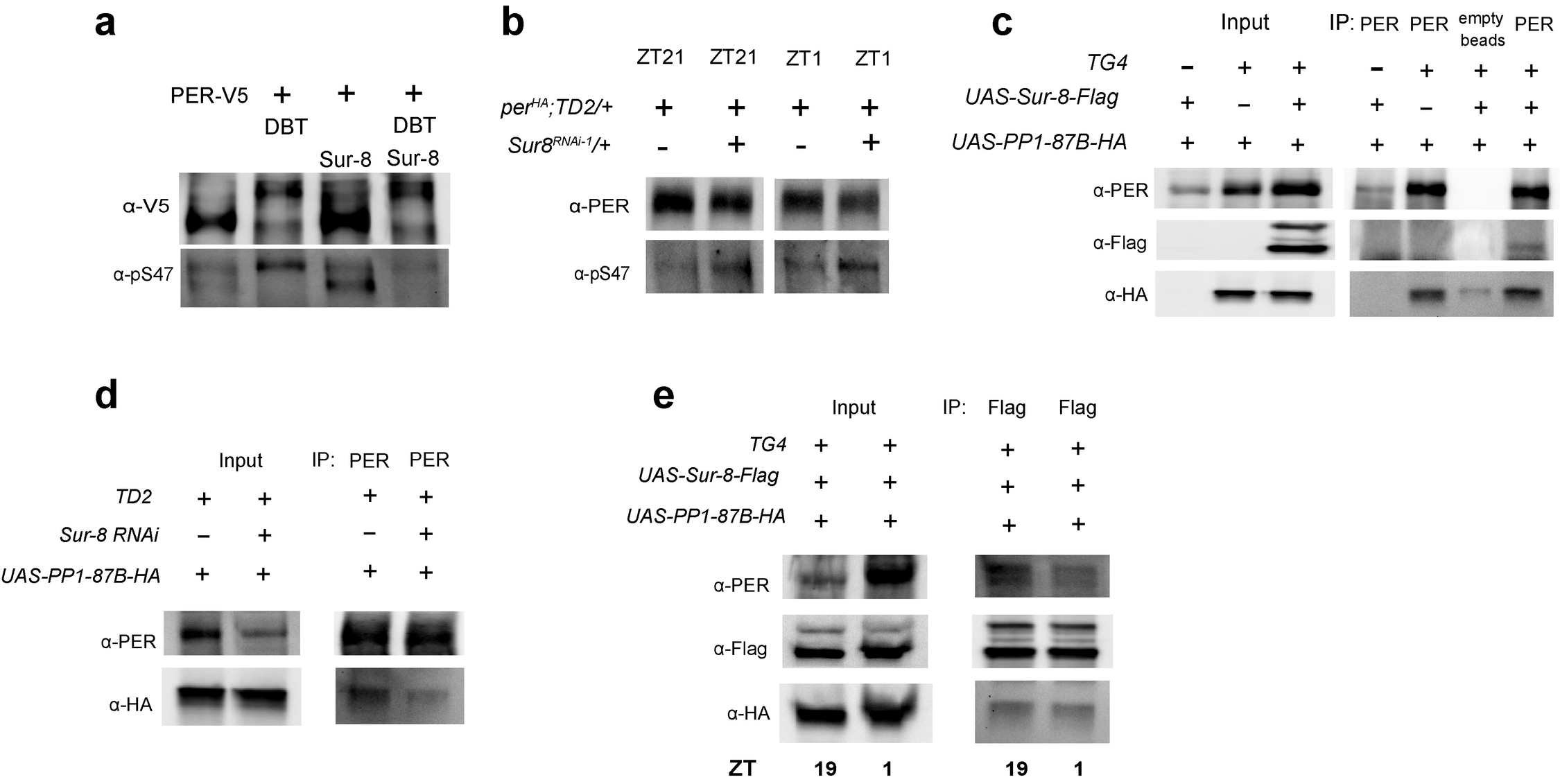

Supplement: S7 Fig — a Additional repeat of (Fig 6E). b Additional repeat of (Fig 6F). c Additional repeat of (Fig 7E). d Additional repeat of (Fig 7G). e Additional repeat of (Fig 7H). (TIF) [file pgen.1008475.s007.tif]
